# Single‐Cell Transcriptomic Profiling Identifies B Cell‐Intrinsic Dysregulation of Sphingolipid Biosynthesis and Could Be Improved by Inhibiting UGCG in Primary Immune Thrombocytopenia

**DOI:** 10.1002/advs.77980

**Published:** 2026-09-27

**Authors:** Dongmei Luo, Yanxia Zhan, Lili Ji, Pengcheng Xu, Pu Chen, Hao Pang, Yunan Ling, Fanli Hua, Xibing Zhuang, Hao Chen, Yunfeng Cheng

**Affiliations:** ^1^ Department of Hematology, Zhongshan Hospital Fudan University Shanghai China; ^2^ Institute of Clinical Science, Zhongshan Hospital Fudan University Shanghai China; ^3^ Department of Clinical Laboratory, Zhongshan Hospital Fudan University Shanghai China; ^4^ Department of Hematology, Zhongshan Hospital Qingpu Branch Fudan University Shanghai China; ^5^ Center for Tumor Diagnosis & Therapy, Jinshan Hospital Fudan University Shanghai China; ^6^ Department of Thoracic Surgery, Zhongshan – Xuhui Hospital Fudan University Shanghai China; ^7^ Shanghai Engineering Research Center for Tumor Multitarget Gene Diagnosis Fudan University Shanghai China

**Keywords:** B cells, primary immune thrombocytopenia, RUNX3, single‐cell RNA sequencing, UGCG

## Abstract

Primary immune thrombocytopenia (ITP) is an autoimmune disease, in which B‐lymphocytes drive autoantibody production and maintain pro‐inflammatory microenvironments. Single‐cell RNA sequencing (scRNA‐seq) datasets were conducted on peripheral blood mononuclear cells samples (4 ITP and 4 thrombocytosis patients from our hospital and 4 healthy donors from GEO database (GSE163668)), and datasets were processed by bioinformatics and machine learning algorithms. Flow cytometry (FCM) was conducted to validate identified cell subsets. UGCG expression in ITP mice was measured using FCM, ELISA, and Ultra‐high liquid lipid mass spectrometry. The UGCG pathway was inhibited. Single‐cell analysis identified a unique B cell subset specifically enriched in ITP patients. SCENIC and hdWGCNA analyses screened core transcriptional regulators including RUNX3, KLF8, and ZNF76, and constructed the RUNX3‐centered regulatory network driving B cell phenotypic transformation. Metabolic profiling revealed aberrantly activated sphingolipid biosynthesis in ITP pathogenic B cells, with UGCG verified as the pivotal hub gene. In vivo experiments confirmed that UGCG inhibition by ibiglustat effectively attenuated platelet clearance in ITP mice. In conclusion, this study identifies a dysregulated sphingolipid metabolic axis in ITP‐specific B cells. Targeting UGCG‐mediated sphingolipid metabolism alleviates thrombocytopenia, revealing a novel B cell‐dependent pathogenic mechanism and providing a promising therapeutic target for ITP.

## Graphical abstract

This study generates the single‐cell atlas of pathogenic B‐cell subset Bc‐5 in ITP. It identifies RUNX3 as a key survival regulator and uncovers sphingolipid‐driven metabolic reprogramming, validating UGCG inhibition as a therapeutic candidate and building a valuable resource for ITP mechanistic and therapeutic exploration.

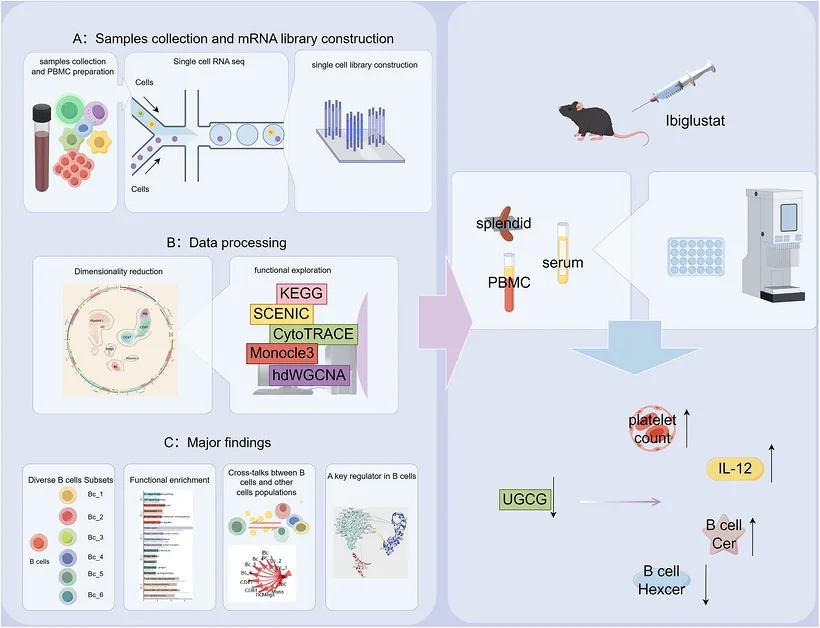

**Abbreviations**
ABCA7: ATP‐binding cassette subfamily A member 7
AICDA: Activation‐induced cytidine deaminase
AIM2: Absent in melanoma 2
ANOVA: Analysis of variance
ATP: Adenosine triphosphate
ATXN2L: Ataxin 2 like
AVMA: American Veterinary Medical Association
BCR: B‐cell receptor
BTK: Bruton tyrosine kinase
CCR5: C‐C motif chemokine receptor 5
CCR6: C‐C motif chemokine receptor 6
CD: Cluster of differentiation
Cer: Ceramide
CIITA: Class II major histocompatibility complex transactivator
CSR: Class‐switch recombination
CTL: Cytotoxic T lymphocyte
CXCR3: C‐X‐C motif chemokine receptor 3
DC: Dendritic cell
DMSO: Dimethyl sulfoxide
EBV: Epstein–Barr virus
ELISA: Enzyme‐linked immunosorbent assay
ET: Essential thrombocythemia
FACS: Fluorescence‐activated cell sorting
FBS: Fetal bovine serum
FCM: Flow cytometry
FPKM: Fragments per kilobase of transcript per million mapped reads
GCS: Glucosylceramide synthase
GEO: Gene Expression Omnibus
GRCh38: Genome Reference Consortium Human Build 38
GTEx: Genotype‐Tissue Expression
HC: Healthy control
hdWGCNA: High‐dimensional weighted gene co‐expression network analysis
HexCer: Hexosylceramide
HISAT2: Hierarchical Indexing for Spliced Alignment of Transcripts 2
ITP: Primary immune thrombocytopenia
JMJD2A: Jumonji domain‐containing protein 2A
KEGG: Kyoto Encyclopedia of Genes and Genomes
KLF8: Kruppel‐like factor 8
LSD: Least significant difference
NK: Natural killer
OD: Optical density
OR: Odds ratio
OXPHOS: Oxidative phosphorylation
PBMC: Peripheral blood mononuclear cell
PEG: Polyethylene glycol
PPI: Protein–protein interaction
PRF1: Perforin 1
qPCR: Quantitative polymerase chain reaction
RFX: Regulatory factor X
RIN: RNA integrity number
ROGUE: Ratio of global unshifted entropy
RUNX1: Runt‐related transcription factor 1
RUNX3: Runt‐related transcription factor 3
SCENIC: Single‐cell regulatory network inference and clustering
scRNA‐seq: Single‐cell RNA sequencing
SD: Standard deviation
SM: Sphingolipid metabolism
SOX4: SRY‐box transcription factor 4
STRING: Search Tool for the Retrieval of Interacting Genes/Proteins
SYK: Spleen tyrosine kinase
TBP: TATA‐box binding protein
TF: Transcription factor
TFIID: Transcription factor IID
Th17: T helper 17
TLS: Tertiary lymphoid structure
TPO‐RA: Thrombopoietin receptor agonist
t‐SNE: t‐distributed stochastic neighbor embedding
UGCG: UDP‐glucose ceramide glucosyltransferase
UMAP: Uniform manifold approximation and projection
WGCNA: Weighted gene co‐expression network analysis
XGBoost: Extreme gradient boosting
XRCC5: X‐ray repair cross‐complementing protein 5
XRCC6: X‐ray repair cross‐complementing protein 6.

## 1 Background

Primary immune thrombocytopenia (ITP) is a multifaceted acquired autoimmune disorder characterized by the production of pathogenic autoantibodies that target platelets, leading to their accelerated destruction and subsequent thrombocytopenia [1, 2, 3, 4]. This condition significantly increases the risk of bleeding and is driven by dysregulated immune responses [5, 6, 7, 8]. Central to this dysregulation are B cells, which play a critical role in generating autoantibodies, perpetuating platelet clearance, and contributing to disease progression.

Some regimens for ITP primarily target B‐cell depletion, including approaches anti‐CD20 therapies [9, 10, 11, 12], Bruton tyrosine kinase (BTK) inhibitors [13, 14, 15], spleen tyrosine kinase (SYK) inhibitors [16, 17, 18], and  CD38^+^ antibody et al. These strategies aim to suppress autoantibody production, disrupt B‐cell signaling, and inhibit platelet destruction [19, 20, 21, 22]. There are patients may still resistance or relapse [23, 24, 25], underscoring the critical need to explore novel treatment strategies. Recent studies have characterized immune cellular populations in ITP patients and animal models [26, 27]. Traditional classifications based on developmental stages, surface markers (e.g., CD19, CD20, CD27) [26, 28, 29], and activation status fail to fully delineate the distinct subpopulations or their specific roles in disease pathology [30]. This gap underscores the need for more refined approaches to dissect the heterogeneity of B cells in autoimmune contexts.

The application of single‐cell transcriptomics in ITP research holds immense promise for uncovering the transcriptional programs governing B cell activation [31], differentiation, and antibody production. By isolating and sequencing individual B cells from ITP patients, rare pathogenic subsets and dysregulated pathways that contribute to the disease can be identified, molecular signatures specific to pathogenic B cells can be revealed, offering potential biomarkers for diagnosis and therapeutic intervention.

To study the cellular changes associated with this prominent behavioral shift and better understand the downstream effects of manipulating the transcription factors (TF) RUNX3, we conducted scRNA seq allying with hdWGCNA [32], to perform an unbiased analysis of the transcriptome, which identified heterogeneity of B cells in ITP and depicted transcriptomic networks regulated by RUNX3 that were related to specific B cell subsets regulation of T cells cytotoxicity in ITP. Elucidating the metabolic characteristics of specific B cells in ITP is essential, as it may reveal novel disease mechanisms and identify metabolically targeted therapeutic strategies.

## 2 Methods

### 2.1 Human Participants and Sample Acquisition and Cell Sorting

For single‐cell RNA sequencing(scRNA‐seq), peripheral blood was collected from newly diagnosed ITP and ET patients. Peripheral blood scRNA‐seq datasets of healthy donor were downloaded from GEO database (GSE163668). The study was approved by the local Medical Ethics Committees of Zhongshan Hospital, Fudan University, and written informed consent was obtained from each participant.

Density gradient centrifugation was used to isolate PBMCs. The isolated PBMCs were viably frozen in fetal bovine serum (FBS) (Gibco, 12483020) with 10 % dimethyl sulfoxide (DMSO) (Sigma–Aldrich, D5879), cryopreserved in liquid nitrogen before being sent for further processing.

### 2.2 Single‐Cell RNA Library Preparation and Sequencing

ScRNA‐seq on PBMC samples using the Drop‐seq method was conducted. The cell suspensions were processed following the 10x Genomics protocol, which creates uniformly barcoded droplets for cell lysis and subsequent library preparation, utilizing the 10x Chromium Controller. Library construction was carried out with the 10x Genomics Single Cell 3’v3 and Chromium Next GEM Single Cell 5’v1 reagent kits. Sequencing was executed on the Novaseq 6000 platform. This approach allowed us to detect the expression of approximately 1000–2000 genes per cell.

The data was analyzed using Cell Ranger v7.0 with the GRCh38 human reference genome sequence. The quality of the data was assessed using the Seurat R package [33], and any cell doublets were removed using the Scrublet R package [34].

Cell‐cycle states of B cells were evaluated using the CellCycleScoring function implemented in Seurat. Canonical S‐phase and G2/M‐phase gene sets from cc.genes.updated.2019 were used to calculate S‐phase and G2/M‐phase scores for each cell. All 43 S‐phase genes and 54 G2/M‐phase genes in the reference gene sets were detected in the dataset and included in the analysis. Based on the resulting scores, individual cells were assigned to G1, S, or G2/M phase. The distribution of cell‐cycle phases and the corresponding S‐ and G2/M‐phase scores were subsequently compared among the six B‐cell subsets (Bc_1–Bc_6). (Table S8 and Figure S7A–D).

### 2.3 Pseudotime Ordering of the Single‐Cell Transcriptome Data

We employed a multi‐algorithmic strategy to reconstruct transitional dynamics among B cell subsets. Pseudotemporal ordering was independently calculated through To infer the trajectory of B cells, it was performed pseudotime ordering of the B cells based on the single‐cell transcriptome data using the R package Monocle3 (v1.3.1, tutorial: https://cole‐trapnell‐lab.github.io/monocle3/) [35] and CytoTRACE (v0.3.3) [36] integration, enabling cross‐validated trajectory mapping of cellular state evolution. Pseudotemporal gene regulatory dynamics were resolved via graph‐autocorrelation algorithms (the “graph_test” implementation), employing tiered significance criteria: transcriptomic trajectories (q‐value < 0.05) were decoupled through Benjamini‐Hochberg adjusted thresholds.

### 2.4 Single‐Cell Regulatory Network Inference and Clustering and Cells Communication

Regulatory landscape reconstruction utilized SCENIC's [37] genome‐wide footprinting to systematically identify transcription factor cooperativity patterns. Motif enrichment was analyzed using paired databases: “hg38__refseq‐r80__10kb_up_and_down_tss.mc9nr.feather” and “hg38__refseq‐r80__500bp_up_and_100bp_down_tss.mc9nr.feather”. Gene coexpression modules within B cells were systematically characterized using network‐based approaches. Modules demonstrating statistically significant TF motif enrichment were selected and designated as regulons. Single‐cell regulon activity quantification was performed, calculating regulatory potential scores for each transcriptional module at cellular resolution. Intercellular communication networks were decoded via complementary ligand‐receptor analysis frameworks (CellChat) [38], focusing on B cells crosstalk with innate and adaptive immune compartments.

### 2.5 High Dimensional Weighted Gene Coexpression Network Analysis

Functional modularization of B cell transcriptomes was achieved through high‐dimensional WGCNA implementation (R package hdWGCNA: V.0.4.05), establishing scale‐free topological frameworks at single‐cell resolution following established tutorial (https://smorabit.github.io/hdWGCNA/articles/basic_tutorial.html) [32]. Cellular module scores were computed at single‐cell resolution. Following establishment of scale‐free topology criteria (>0.85), a soft threshold power of 5 was optimized for network connectivity. protein–protein interaction networks were subsequently reconstructed using the HubGeneNetworkPlot algorithm.

### 2.6 Bulk RNA‐Seq Sequencing and Data Analyses

Total RNA extraction was carried out from 4 ITP patients’ PBMCs with Trizol reagent (Invitrogen) according to the manufacturer's instructions. Upon qualification by NanoDrop 2000 (OD260/280: 1.6‐1.8) and Agilent 2100 (RIN> 7.0), sequencing libraries were constructed and analyzed on an Illumina NovaSeq 6000 system (CFBiotech) as per the standard sequencing protocol.

Quality control of the raw sequencing data was conducted using Trimmomatic (v.0.39) [39], with subsequent quality assessment performed by FastQC (v.0.11.9). Further processing of the reads was carried out with fastp (v.0.22.0). Alignment of the processed Fastq files to the GRCh38 human genome was achieved through HISAT2 (v.2.1.0). Gene expression quantification in both read counts and FPKM was generated by StringTie (v.2.1.3b). Healthy control data for comparative analysis were sourced from the GTEx database(https://www.gtexportal.org/home/aboutAdultGtex).

### 2.7 Mice Treatment by Ibiglustat

C57BL/6J mice (ages 8–12 weeks) were divided into three experimental groups: WT + ibiglustat, Treat, and Placebo. Mice in the WT + ibiglustat group were not subjected to CD41 antibody‐induced ITP modeling and received ibiglustat alone. Mice in the Treat and Placebo groups were subjected to CD41 antibody‐induced ITP modeling as previously described [40]. The Treat group received ibiglustat at 12.5 mg/kg/day by intraperitoneal injection, whereas the Placebo group received an equivalent volume of vehicle by the same route. Ibiglustat was prepared in a vehicle containing polyethylene glycol and Tween‐80. Blood counts were measured on days 0, 2, 4, and 6. All mice were sacrificed on day 7, and spleen tissues and peripheral blood were collected for subsequent analyses. Prior to euthanasia, mice were anesthetized with inhaled isoflurane (induction concentration approximately 4%, maintenance concentration approximately 1.5%–2%). Once the animals were fully unconscious and showed no response to pain or reflex stimuli, cervical dislocation was performed by personnel trained and proficient in animal experimentation techniques. This method complies with the American Veterinary Medical Association (AVMA) Guidelines for the Euthanasia of Animals (2020) for euthanasia of mice. The experiments were approved by the Institutional Animal Care and Use Committee of ZhongShan Hospital.

### 2.8 Mice B Cell Isolation and Purification and Mass Spectrometry

Murine splenic B cells were isolated by positive selection using CD19 MicroBeads (Miltenyi Biotec, 130‐121‐301). The purity of the isolated B cells determined by flow cytometry (on FACSymphony A5 (BD Biosciences) or Aurora (Cytek), consistently exceeded 95%. Sorting was performed on FACSAria Fusion (BD Biosciences). These purified cells were processed for mass spectrometry, and the acquired raw values were normalized based on phosphate concentration.

### 2.9 Flow Cytometry Analysis

Surface marker staining was performed using a panel of fluorescently conjugated antibodies (Table S7), with human B cells being stained for CD19 (PE) and SOX4 (Alexa Fluor 488), respectively. For SOX4 detection, cells were first stained for the surface marker CD19, followed by fixation and permeabilization for intranuclear staining. The fixed and permeabilized cells were subsequently incubated with the anti‐SOX4 antibody, washed with permeabilization buffer, and subjected to flow‐cytometric acquisition and analysis. Data acquisition was carried out on either a BD Biosciences flow cytometer or a Cytek Aurora/NL system, and all flow data were analyzed using FlowJo (BD Biosciences).

### 2.10 Statistical Analysis

Statistical analyses were performed using R (version 4.1.0). The Data were expressed as mean ± standard deviation (SD). Continuous measures were compared between groups with the Wilcoxon rank‐sum test, while categorical variables were analyzed using the χ^2^ test. One‐way analysis of variance (ANOVA) was used for comparisons between more than two groups, followed by the least significant difference (LSD) multiple comparison test. *P* value < 0.05 was considered to indicate statistically significant.

## 3 Results

### 3.1 Single‐Cell Transcriptomes of B Cells

**FIGURE 1:**
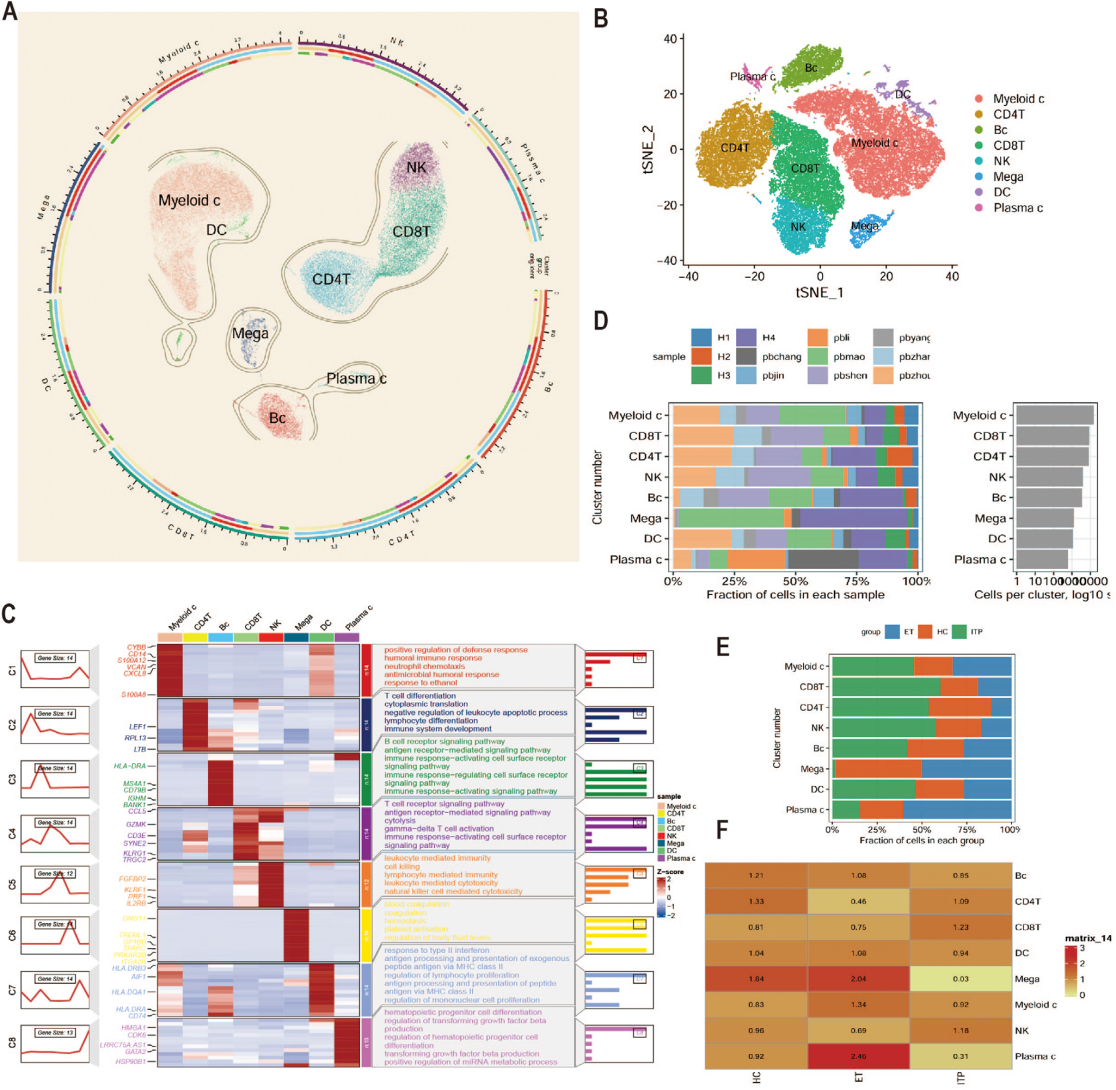
Single‐cell profiling of Peripheral Blood Mononuclear Cells across. (A) Uniform manifold approximation and projection (UMAP) visualization of 8 cell clusters. (B) TSNE visualization of 8 cell clusters. (C) ComplexHeatmap for the expression of marker genes in subsets. Colors represent maximum‐normalized mean expression of marker genes. (D,E) Stacked bar plots showing the subsets distribution in ET, ITP, and HC. (F) Heatmap showing the odds ratios (ORs) of subset distribution in ET, ITP, and HC. The OR value represents the preference distribution in ET, ITP, and HC.

ScRNA‐seq was conducted on PBMCs from 4 ITP patients and 4 ET patients, healthy controls (HCs) data were obtained from the GEO database. The characteristics of the participants are summarized in Supplemental tables. Following quality control and batch effect correction, a total of 43 356 single‐cell profiles were retained for downstream analysis, comprising 10 633 cells from ET patients, 11 396 cells from HCs, and 21 327 cells from ITP patients. Dimensionality reduction using uniform manifold approximation and projection (UMAP) demonstrated effective integration of the datasets across different sample groups (Figure 1A,B; Figures S1A and S8A–D and Table S9). The proportions of each cell population across groups are shown in Figure 1D,E. We further analyzed the tissue preferences of these cell populations, as shown in Figure 1F.

Using characteristic cell markers, we identified diverse cell populations, including myeloid cells (marked by S100A8, CXCL8, and CYBB), megakaryocytes (GNG11 and ITGA2B), natural killer (NK) cells (PRF1, KLRF1, and IL2RB), dendritic cells (DCs, CD74 and AIF1), B cells (IGHM, CD79B, and MS4A1), plasma cells (HMGA1 and CDK6), CD4^+^ T cells (LTB and LEF1), and CD8^+^ T cells (GZMK, CCL5, and TRGC2) (Figure 1A,C).

### 3.2 Construction of a B Cells Single‐Cell Atlas in ITP

**FIGURE 2:**
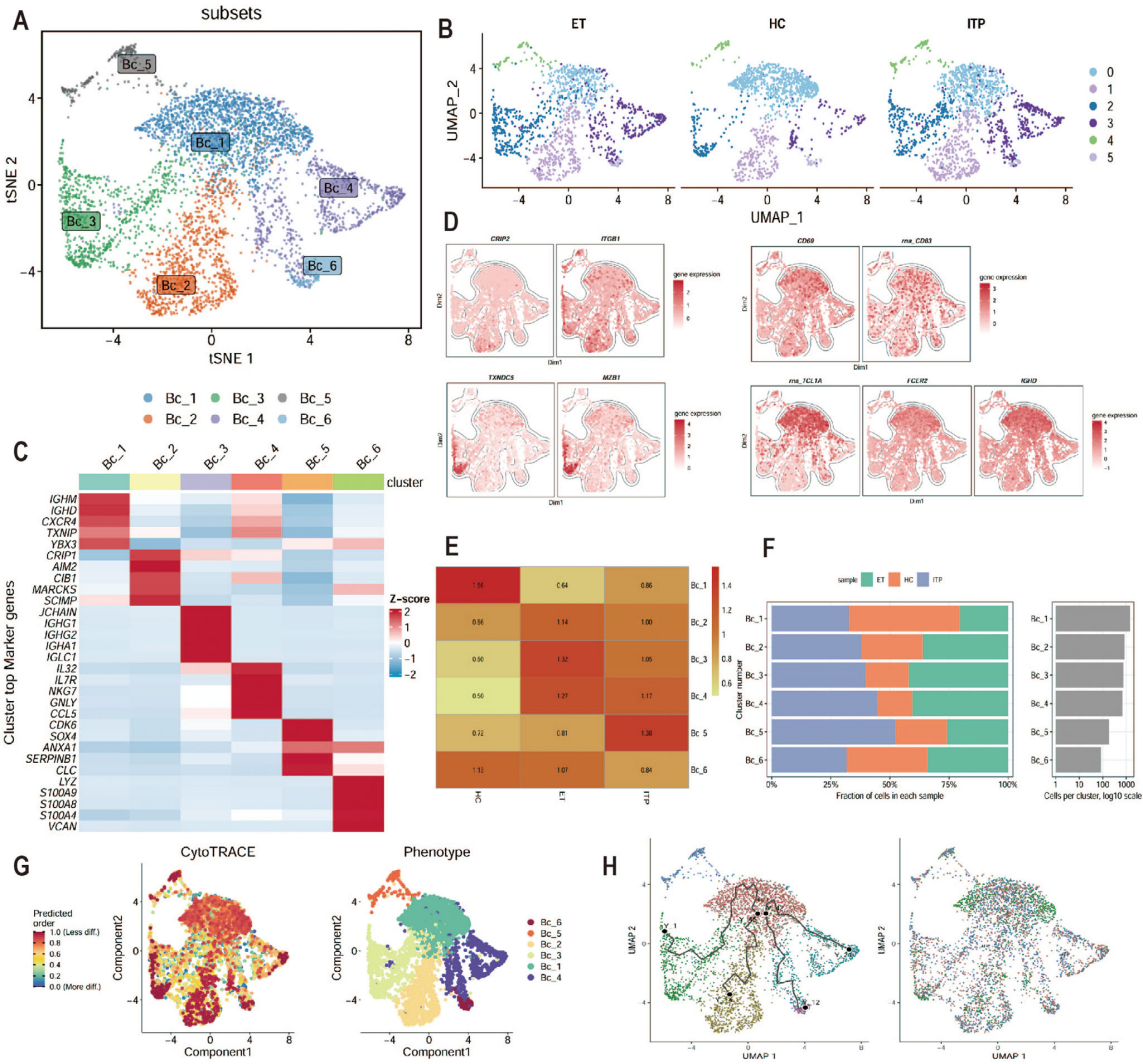
Identification of B cell subsets in ITP. (A) Uniform manifold approximation and projection (UMAP) visualization of 6 B cell subsets. (B) Uniform manifold approximation and projection (UMAP) visualization of 6 B cell subsets in different groups. (C) Heatmap illustrates the differentially expressed genes among the 6 B cell subtypes. (D) UMAP visualization of the signatures of Atypical memory (ITGB1 and CRIP2), activated B cells (CD69 and CD83), antibody‐secreting cells(TXNDC5 and MZB1), and naïve B cell (TCL1A, FCER2, and IGHD). (E) Heatmap showing the odds ratios (ORs) of B cell subset distribution in each group. The OR value represents the preference distribution in corresponding group. (F) Stacked bar plots showing the subset distribution in the corresponding group within total B cells. (G) UMAP plots showing the identification of B cell subsets(right), development order by CytoTRACE (left). (H) UMAP plots showing the B cells differentiation state inferred by Monocle3(left); differentiation state group by diseases(right), Healthy control was colored by green;ITP was color by blue; ET was colored by red.

All scRNA‐seq datasets for B cells and plasma cells were integrated after removing batch effects using the Integration algorithm. Systematic unsupervised clustering was performed to define transcriptional subsets of B cells. Six major clusters were identified, encompassing five B cell lineages Bc‐1, Bc‐2, Bc‐4, Bc‐5, and Bc‐6, along with plasma cells. Independent analyses of individual disease types yielded consistent clustering results (Figure 2A,B). The quantify of transcriptomic purity of identified B cell subsets by ROGUE analysis [41], indicate high gene expression heterogeneity among distinct subclusters (Figure S1B).

Using gene set‐based analyses, Bc‐1 exhibited transcriptional phenotypes similar to naïve B cells (IGHM, IGHD, and YBX3), while Bc‐2 aligned with memory B cells (AIM2, CIB1, MARCKS, and SCIMP). Bc‐3 displayed transcriptional signatures consistent with plasma cells (JCHAIN, IGHA1, IGHG1, IGHG2, and HSPA5) (Figure 2D; Table S2). Most excitingly, three previously uncharacterized subsets were identified: Bc‐4, which highly expressed IL32, IL7R, NKG7, and GNLY, associated with active B cells; Bc‐5, marked by elevated expression of CDK6, SOX4, ANXA1, CLC, and SERPINB1, linked to T cell lineage development; and Bc‐6, characterized by high expression of LYZ, S100A8, S100A9, and VCAN, associated with inflammation and autophagy (Figure 2C). To assess whether Bc_5 represents a proliferative or cycling B‐cell population, we performed cell‐cycle scoring across all B‐cell subsets. Bc_5 comprised 57.0% G1‐, 26.4% S‐, and 16.6% G2/M‐phase cells, with no apparent enrichment of cycling cells or elevated S/G2M scores compared with other major B‐cell subsets. These findings suggest that the distinct transcriptional features of Bc_5 are unlikely to be primarily driven by cell‐cycle activity (Figure S7A–D and Table S8).

Through subset distribution analysis, we observed that ITP exhibited the highest proportion of Bc_5 cells, whereas Bc_1 cells were predominantly found in HCs (Figure 2F). Odds ratio (OR) analysis revealed that Bc‐5 cells were strongly associated with ITP, while Bc_2, Bc_3, and Bc_4 subsets were significantly enriched in ET. In contrast, Bc_1 cells were predominantly enriched in HCs (Figure 2E).

**FIGURE 3:**
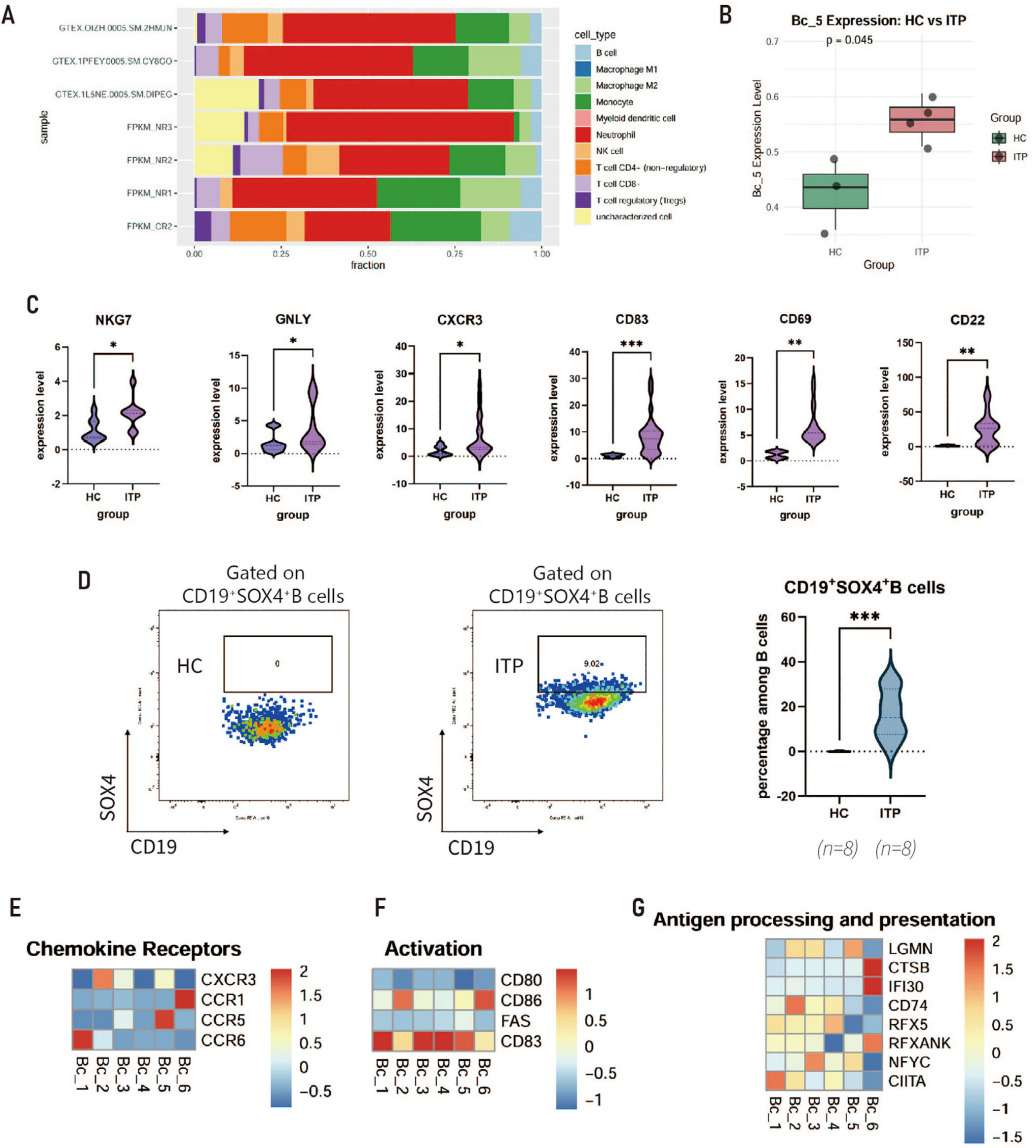
Confirmation of SOX4^hi^ CD19^+^ B cell subsets in ITP. (A) Stacked bar plots showing the immune cells distribution in the bulk‐RNAseq of ITP patients (n = 4) and healthy controls (n = 3). (B) Boxplot showing the expressing levels of SOX4^hi^ CD19^+^B cells in bulk RNA‐seq of ITP patients (n = 4) and healthy controls (n = 3). (C) Violin plots showing the expression level of NKG7, GNLY, CXCR3, CD83, CD63 and CD22 in PBMC of healthy donors and ITP patients. (D) FACS plots and percentage of SOX4^hi^ CD19^+^ B cells from peripheral blood; Representative flow cytometry plots from HC and ITP groups. Violin plots showing the frequency of SOX4hiCD19+ B cells in HC and ITP samples. (E–G) heatmap showing functional pathways enriched in B cells subsets. (E) genes relating to Chemokine receptors; (F) genes relating to B cell activation. (G) genes relating to antigen processing and presentation.

The further analyzed data showed that Bc_1, Bc_2, Bc_3, Bc_4, and Bc_5 exhibited robust activation (Figure 3C, F). Chemokine receptor profiling revealed subset‐specific enrichment patterns (Figure 3E): Bc_1 cells demonstrated marked enrichment of CCR6, Bc_2 cells exhibited CXCR3 predominance, and Bc_5 cells showed significant CCR5 expression (Figure 3E). In contrast, no chemokine receptor enrichment was detected in Bc_3 or Bc_4 subsets. Transcriptional analysis during antigen processing and presentation highlighted distinct regulatory signatures: Bc_1 upregulated CIITA and RFX5, Bc_2 displayed elevated CD74 expression, Bc_3 showed NFYC activation, Bc_4 was characterized by RFX4 induction, and Bc_5 exhibited increased LGMN levels (Figure 3G). qPCR analysis of PBMCs from ITP patients revealed elevated expression of B cell activation‐related genes (CD83 and CD69) (Table S1). We calculated class‐switch recombination (CSR) [42] scoring (CSR‐related signature score and genes: *APEX1*, *APEX2*, *XRCC5*, *XRCC6*, *POLD2*, and *AICDA*) [43] on B cells subsets to figure out if any B cell populations were undergoing CSR. Results demonstrated significantly elevated CSR scores in ITP patients relative to HC, while no difference was observed between Bc_1 cells and Bc_5 cells (Figure S1D,E).

To infer the differentiation state of B cells in ITP and to understand the source of Bc_5 cells in ITP, two trajectory analysis methods were applied, comprising CytoTRACE [36] and Monocle3 [35]. CytoTRACE algorithm data suggested that the pseudotime ordering recapitulated the phenotypic modulation of B cells, which showed less differentiation in Bc_1 cells compared with other B cells subsets (Figure 2G). Monocle3 analysis confirmed this (Figure 2H; Figure S2A). By utilizing Monocle3, the change in pseudotime of marker genes within different B cells subclusters were consistent with the differentiation state of B cells, reflecting their potential role in phenotypic switching (Figure S2B–I).

Bulk‐seq data by CIBERSORT also showed an increase in B‐cell proportion in ITP patients compared to HCs (Figure 3A; Figure S6B). Deconvolution analysis of the Bulk‐seq data with markers of BC_5 cells (*CDK6*, *SOX4*, *ANXA1*, and *CLC*) revealed significant enrichment of the BC_5 cell subset in ITP patients compared to HCs (Figure 3B). The flow cytometric analysis results from another independent cohort further confirmed the presence of SOX4^hi^CD19^+^ B cells, and the frequency of this specific subpopulation was significantly higher in patients with ITP than in healthy controls. (Figure 3D; Figure S6A)

### 3.3 Cellular Interaction between SOX4^hi^CD19^+^ B Cells and T Cells was Depicted

**FIGURE 4:**
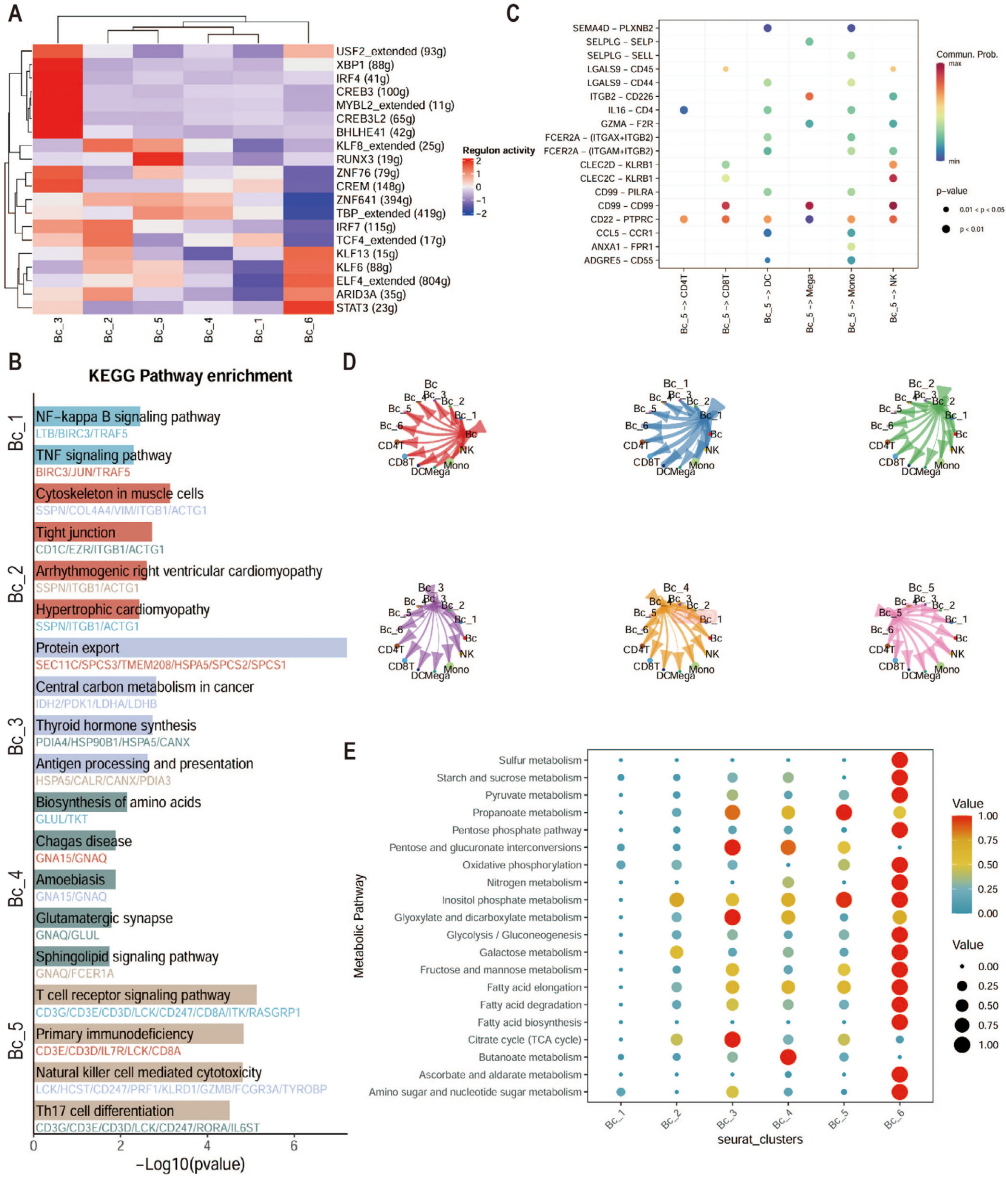
Cross‐talk between SOX4^hi^ CD19^+^ B cell subsets and other immune cells. (A) Heatmap of TF activity by SCENIC of the annotated B cell subsets. (B) The KEGG enrichment analysis of differentially expressed genes in B cell subsets. (C) Dot plot visualized the result of Cellchat showing the primary pathways of communication between SOX4^hi^ CD19^+^B cells and other cells. (D) Chord plot showing cell communication in the B cell subsets and other cell clusters. (E) Dot plot visualized the result of scMetabolism showing the upregulation metabolism pathways in B cell subsets.

The results of KEGG pathway analysis of functional pathways associated with B cell subsets indicated that Bc_5 cells were enriched in pathways related to T cell receptor signaling, natural killer cell‐mediated cytotoxicity, and Th17 cell differentiation (Figure 4B; Figure S1C). The genes predominantly expressed in B cells were enriched in T cell receptor signaling and regulation of cytotoxic T cell‐related pathways, there might have potential bidirectional crosstalk between specific B cell subsets and T cells in ITP. Cell‐cell communication networks were investigated in ITP through CellChat [44] algorithm, focusing on ligand‐receptor interactions and functional coordination between B cell subsets and T cells subpopulations. The data showed a number of interactions between T cells and B cells (Figure 4C). Ligand‐receptor analysis suggested that interactions involving *TIM3*, *LGALS9* and *FLT3* were low probability, and suggested that interactions mediated by *CD22‐PTPRC* were present in the cellular communication of SOX4^hi^CD19^+^ B cells with CD4^+^T cells, CD8^+^Tcells, DCs and NK cells, while *CD99‐CD99* axis was detected among the interactions of SOX4^hi^CD19^+^ B cells with CD8^+^T cells, megakaryocyte and NK cells (Figure 4D). These axis with mentioned populations were exclusively observed in SOX4^hi^CD19^+^ B cells (Figure S3A–D). Collectively, these findings have revealed the involvement of specific signaling molecules in the communication between B cells, T cell, and megakaryocyte in ITP patients.

### 3.4 RUNX3 Driving the Phenotypic Modulation of B cells in ITP

TFs enriched in the implicated cell types were explored to understand the regulatory mechanisms involved in expansion of SOX4^hi^CD19^+^ B cells in ITP. The epigenomic landscape of ITP was analyzed by scRNA‐seq and using the SCENIC algorithm. TFs enriched across different B cell subsets were identified, revealing both shared and distinct regulatory patterns (Figure 4A; Table S6). KLF8, a TF involved in DNA damage and cell cycle regulation, exhibited shared activity in both Bc_2 and SOX4^hi^CD19^+^ B cells subsets. RUNX3, a TF critical for T cell cytotoxic phenotype acquisition, was specifically expressed in the SOX4^hi^CD19^+^ B cells subset, suggesting its potential role in modulating T cell activity in ITP.

**FIGURE 5:**
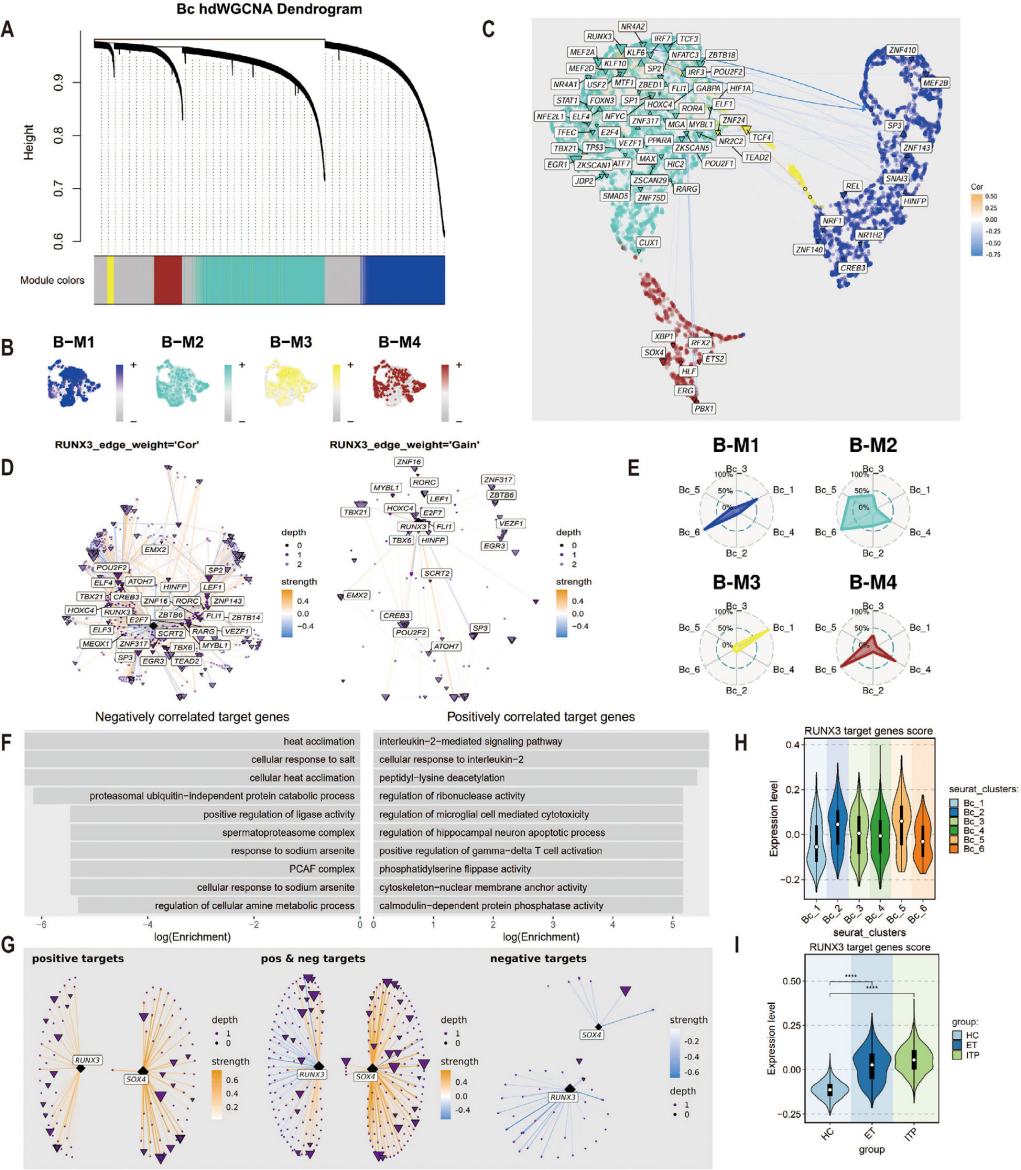
Candidate key regulators potentially driving the phenotypic modulation of ITP and the gene co‐expression modules among SOX4^hi^ CD19^+^B cells. (A) Dendrogram showing the coexpression modules of the ITP B cells identified by hdWGCNA. (B) UMAP plots showing the expression distribution of 4 modules across all B cell subsets. (C) Co‐expression UMAP showing the RUNX3 TF regulatory network, genes were colored according to their co‐expression module assignment. (D) RUNX3 regulatory network in B cells. Nodes represent individual TF and are colored based on their relationship with RUNX3. Directed edges represent TF regulatory relationships, colored by TF‐gene gene expression correlation. Left based on TF‐gene Pearson correlation (edge_weight='Cor'), right based on the importance estimated by the XGBoost model. Selected TFS are shown as diamonds, other TFS are shown as triangles, and genes are shown as circles. The size of each node corresponds to the out‐degree (number of outgoing connections) in the network. The color of the edge indicates the strength of the TF‐gene interaction based on the Pearson correlation of gene expression. (E) Radar plot visualizing the relative expression level of each module across different B cell subclusters. (F) Selected pathway enrichment results for RUNX3 target genes identified in B cells. Results shown for target genes with negative (left) and positive (right) gene expression correlations with RUNX3. (G) TFNetworkPlot shows a network plot with selected TFs SOX4 and RUNX3. Target_type shows different plots for the positive and negative TF‐gene relationships. (H,I) violin plots of the scRNA‐seq dataset colored by gene expression scores for RUNX3 target genes in B cells. (H) group by B cells subset; (I) grouped by diseases (*p* < 0, 0.001, 0.01, 0.05, 1, symbols ="****","***", "**", "*", "ns”.).

To uncover key regulators influencing the phenotypic changes in the SOX4^hi^CD19^+^ B cells subset, we conducted high‐dimension single‐cell weighted gene co‐expression network analysis (hdWGCNA) on B cells. This analysis revealed four gene co‐expression modules (Figure 5A,B; Figure S4A–E), with one dominant module (B‐M2) showing a strong correlation with SOX4^hi^CD19^+^ B cells (Figure 5E). RUNX3 was upregulated in the B‐M2 module. Potential RUNX3 target genes were further identified, and its regulatory networks were constructed, providing insights into the TF regulatory networks specific to SOX4^hi^CD19^+^ B cells. This conclusion validated the results of the SCENIC analysis. The results of TFNetworkPlot analysis revealed a strong regulatory interaction between SOX4 and RUNX3 (Figure 5G), suggesting that SOX4 may be a direct target of RUNX3.

TF‐gene Pearson correlation was combined with an ensemble learning algorithm, extreme gradient boosting trees (XGBoost) to identify key TF‐gene interactions, to model gene expression based on potential TF regulators. XGBoost allowed us to compute TF regulatory scores, which quantify the contribution of each TF to the expression of its target genes, thereby identifying the most likely regulatory relationships. The directionality of these scores was determined by the co‐expression patterns between TFs and their potential target genes (Figure 5C,D; Figure S5 and Table S5). ABCA7 is known to mediate the transport of phospholipids across cell membranes [45] and ATXN2L has been identified as a novel regulator of stress granules [46]. Composite gene expression scores (AddModuleScore) were calculated for RUNX3 target genes across B cell subsets and different groups. Our data suggests RUNX3's broad regulatory impact in SOX4^hi^ CD19^+^ B cells and its association with ITP (Figure 5H,I; Figure S4C,E).

Pathway enrichment analysis was performed in the sets of RUNX3 primary target genes (Figure S5), to understand which biological processes are potentially regulated by RUNX3 in the SOX4^hi^CD19^+^ B cells, stratifying by activating and repressing target genes (Table S6). The results showed that RUNX3 primary target genes were significantly enriched in pathways related to critical inflammatory signaling and T cell regulation, including interleukin‐2‐mediated signaling pathway, cellular responses to interleukin‐2, and positive regulation of gamma‐delta T cell activation (Figure 5F; Figure S4C). Gene co‐expression modules associated with the mechanism among B cells of ITP were identified in the study.

### 3.5 UGCG Drives Sphingolipid Metabolism Disorder in SOX4^hi^CD19^+^ B Cells

**FIGURE 6:**
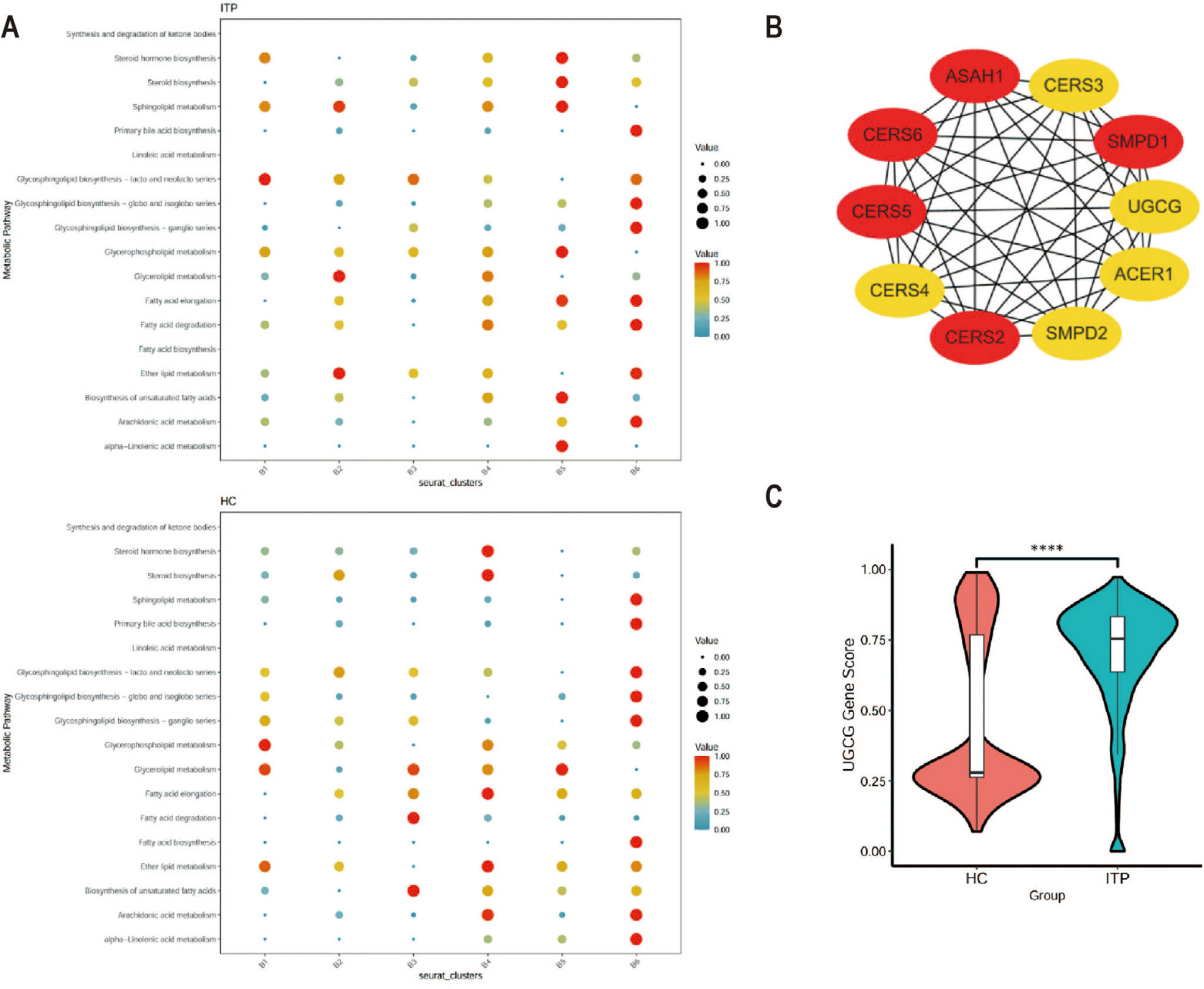
UGCG is upregulates in SOX4^hi^ CD19^+^ B cells. (A) Dot heatmaps showing the enrichment of metabolism pathways in B cell subsets. (B) Protein–Protein Interaction Network showing key genes in sphingolipid metabolism pathway. (C) Violin plots showing the gene score of UGCG in B cells of healthy donors and ITP patients.

We examined what metabolism factors mechanistically drives the expansion of SOX4^hi^CD19^+^ B cells in ITP patients. The metabolic pathway scores of B cell subsets using scMetabolism and unsupervised clustering of metabolism‐associated genes were computed. Pathway analysis of B cells revealed a strong variance of metabolic pathways across different groups, suggesting the potential metabolic regulation of B cell identity (Figures 4E and 6A; Table S4). Sphingolipid metabolism (SM) was up‐regulated in Bc_5 cells in ITP patients. UGCG was then identified as the hub gene by Cytoscape and showed similar trends with SM (Figure 6B,C).

**FIGURE 7:**
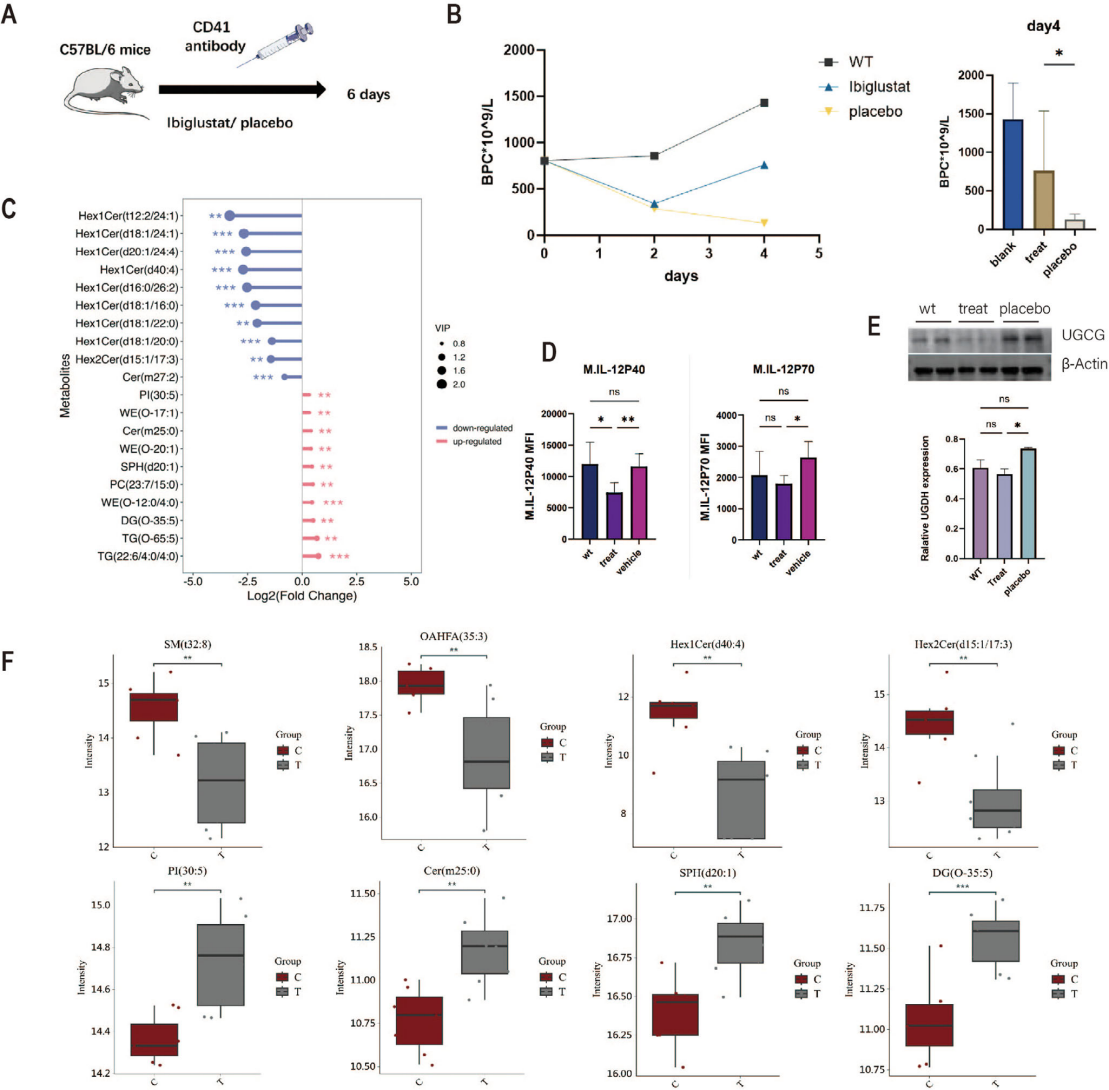
Inhibiting UGCG activity disrupts sphingolipid synthesis in SOX4^hi^CD19^+^B cells and ameliorates thrombocytopenia in ITP. (A) Experimental design. C57BL/6J mice were assigned to a WT + ibiglustat group without CD41 induction, a CD41‐induced ITP group treated with ibiglustat, and a CD41‐induced ITP group receiving vehicle. Ibiglustat or vehicle was administered by intraperitoneal injection for up to 6 days. Mice were sacrificed at the indicated times, and splenic B cells were assessed. (B) Longitudinal changes in platelet counts of C57 mice stimulated with CD41 antibody during ibiglustat or placebo treatment. (C) Lollipop plots illustrating the alterations in B cell sphingolipid metabolism following ibiglustat treatment. (D) Serum inflammatory cytokine levels following UGCG inhibition. Concentrations of IL‐12P40 and IL‐12P70 were quantified by ELISA. Data are presented as mean ± SD. (E) Representative Western blot analysis of UGCG expression in splenic B cells from wild‐type mice receiving ibiglustat alone (WT + ibiglustat), CD41‐induced ITP mice treated with ibiglustat (Treat), and CD41‐induced ITP mice receiving vehicle (Placebo), together with densitometric quantification normalized to β‐actin. (F) Validation of representative lipid metabolites altered following UGCG inhibition. Box plots show relative abundances of SM(32:8), OAHFA(35:3), Hex1Cer(d40:4), Hex2Cer(d15:1/17:3), PI(30:5), Cer(m25:0), SPH(d20:1), and DG(O‐35:5) in control and treatment groups. Data are presented as mean ± SD unless otherwise indicated. Statistical analyses were performed using one‐way ANOVA followed by multiple‐comparison testing or unpaired two‐tailed Student's t test as appropriate. ^*^ *p* < 0.05, ^**^ *p* < 0.01, ^***^ *p* < 0.001; ns, not significant.

The UGCG inhibitor (Ibiglustat) was intraperitoneally injected into CD41 antibody‐stimulated mice to investigate the mechanism of UGCG in ITP (Figure 7A). Platelets number in the Ibiglustat‐treated mice improved to baseline levels compared to the placebo group on the fourth day (Figure 7B; Table S3). After 6 days of treatment, the mice were euthanized on the seventh day. ELISA analysis showed that ibiglustat treatment had no effect on the levels of inflammatory factors in mice serum (Figure 7D; Figure S6C,D). Analysis of UGCG expression in mouse splenocytes revealed its context‐dependent role in ITP. While no significant difference was found between the ibiglustat‐only group (WT+ ibiglustat) and the placebo‐treated CD41‐stimulated group, UGCG expression was specifically reduced in CD41‐stimulated ITP model mice upon ibiglustat treatment (Figure 7E; Figure S6E). This indicates that UGCG inhibition alleviates thrombocytopenia in the ITP model without affecting normal conditions. Splenic B cells were isolated from the mice for mass spectrometry–based untargeted lipidomics. Compared to the placebo group, the treatment group has lower SM and HexCer and higher Cer (Figure 7C,F). This suggests that targeting UGCG has potential therapeutic effects for ITP.

## 4 Discussion

ITP‐related T and B cells serve as essential and synergistic components within the immune environment. Recent investigations have underscored the importance of elucidating T cell states and compositions across various diseases, revealing substantial plasticity in response to stress and autoimmune reactivity [47]; however, the transcriptional heterogeneity of ITP related‐B cells has been underestimated, leaving their controversial roles across different cancer types. In this study, we systematically charted B cells in ITP patients by deciphering their transcriptomics. Strikingly, the validated presence of stressed B cells, which shared similarities with the recently discovered B cell subsets. Our study revealed diverse B cells differentiation pathways and the ITP preference of these pathways. Such preference can be attributed to the maturity of TLSs and metabolic factors.

B cells are high degree of regulatory heterogeneity among different states diseases. previous researches conducted single cell atlas of B cells on pan‐cancer, supporting research into the role of B cells in ITP pathogenesis and treatment [48, 49]. In contrast to previous single‐cell/BCR transcriptome studies focusing on the B cell from bone marrow of ITP patients [50, 51], this study fills the knowledge gap caused by the lack of single‐cell transcriptome datasets of the peripheral blood B cells in ITP, although an BCR‐seq dataset of ITP PBMCs wasn't contained in this study. Some current studies utilizing single‐cell sequencing focus on investigating more accessible cell populations such as neutrophils, macrophages, and T cells, while rarely prioritizing the analysis of B cells. Our study provided a unique and invaluable dataset that is expected to deepen our understanding of ITP related B cells with, for example, the interpretation of risk variants identified by the ever‐growing number of genome‐wide association studies.

Leveraging the scRNA‐seq datasets, we, for the first time, disentangled the complex dynamics of gene expression and transcription factors accessibility during the phenotypic modulation of ITP related B cells. The Bc_5 signature exhibited the strongest association with the expansion among all B cell subsets in ITP. Furthermore, based on the evidence from multiple comparative analyses, such as hdWGNCA, regulon expression activity analysis, TF bind motif activity analysis, SCENIC analysis and pseudotime trajectory analysis, we obtained 3 candidate key regulators that potentially drive the phenotypic modulation of B cells and the pathogenesis of ITP. KLF8, a zinc finger‐containing transcriptional regulator from the Krüppel‐like factor family (C2H2 structural architecture), demonstrates functional dependence on epigenetic coordination with JMJD2A – specifically through tripartite zinc finger‐mediated molecular partnerships that facilitate chromatin remodeling processes. This protein complex modulates the transcriptional activity of cell cycle regulators p21 and CDK4, thereby enhancing mitotic progression and cellular clonogenic capacity [52]. In this research, the upregulation of KLF8 expression may facilitate the expansion of Bc_5 cells in ITP. TATA box‐binding protein (TBP) is a subunit of the multiprotein complex transcription factor (TF) IID, which is important for promoter recognition [53]. We found that the transcription factor TBP may also function as a key regulator for the expansion of Bc_5 cells in ITP.

The reciprocal expression pattern suggests RUNX3 may contribute to the sustained survival of Bc_5 cell populations in ITP pathogenesis through regulatory interactions with RUNX1 (Figure SD‐E). RUNX3 is known essential for T cells acquiring a cytotoxic phenotype [54]. Here we present the first sequencing dataset delineating how RUNX3 perturbation influences its downstream transcriptional network in ITP‐related B cells. Of particular interest, diminished RUNX1 levels appear associated with accelerated B cell proliferation dynamics. Current studies propose that RUNX3 may facilitate Epstein‐Barr virus‐induced B cell proliferation and transformation through suppression of RUNX1 expression [55, 56]. Consistent with these observations, our data showed that decreased RUNX1 expression concomitant with elevated RUNX3 levels in ITP patients. This investigation provides novel evidence identifying RUNX3 as a critical modulator sustaining pathogenic B cell populations in ITP. One recent study demonstrated that RUNX3 orchestrates the differentiation of effector and memory T cells by modulating the expression of key chemokine receptors, particularly CCR3 and CCR5 [57]. In this study, the SOX4^hi^CD19^+^ B cells subset demonstrated the strongest association with CD8^+^ T cells and exhibited elevated RUNX3 expression. Therefore, SOX4hiCD19+ B cells may alleviate the exhaustion of CD8+ T cells in ITP through RUNX3.

Research in immunometabolism has established that key metabolic enzymes exert cell‐subset‐specific functions, which are precisely modulated according to the activation status of immune cells [4, 58]. SOX4^hi^CD19^+^ B cells displayed metabolism reprograming by increase the enrichment of sphingolipid metabolism in ITP. And upregulated sphingolipid biosynthesis, with network topology analysis (STRING/Cytoscape) pinpointing UGCG (glucosylceramide synthase) as a critical hub gene. By inhibiting UGCG with ibiglustat, sphingolipid metabolism of B cells was reduced and platelet count was elevated in ITP mice. Our findings point to an association between B cell expansion and the upregulation of the sphingomyelin in ITP, which is strongly linked to UGCG overexpression. Our data support the potential of UGCG as a viable therapeutic target in ITP.

The study has its limitations. Only new diagnosed ITP patients were enrolled in this study. The generality of the findings to other conditions of ITP, for example, refractory ITP, warrants further investigation. While our data provided mechanistic insights into B lymphocyte‐mediated pathogenic contributions in ITP, the investigation of individual variations in cellular composition was not encompassed within the current research scope. A larger sample size in future studies would enhance the ability to detect potential compositional differences in other cell types.

In summary, we presented the first atlas of gene expression accessibility in ITP at single‐cell resolution. This unique dataset provides novel insights into the regulatory dynamics during B cells modulation in ITP patients. RUNX3 was identified as a novel key regulator for maintaining the survival of specific B cell populations in ITP, which may serve as a potential therapeutic target. Our datasets are expected to serve as a valuable reference to further decipher the regulatory mechanism of ITP. B cells in ITP undergo metabolic reprogramming driven by sphingolipid pathway upregulation, wherein targeting the hub gene UGCG represents a viable therapeutic strategy.

## Author Contributions

**Dongmei Luo**, **Yanxia Zhan**, Pu Chen, **Lili Ji**, **Hao Pang**, **Pengcheng Xu**, **Yunfeng Cheng**, and **Hao Chen** performed the literature review, designed research, drafted and revised the manuscript; Yunfeng Cheng, and Hao Chen contributed to the critical revision of the manuscript; Dongmei Luo, Yanxia Zhan, Lili Ji, Hao Pang, Pengcheng Xu, Yunan Lin, Pu Chen, **Fanli Hua**, **Xibing Zhuang**, and Yunfeng Cheng performed the experiments, analyzed data. All authors read and approved the final manuscript.

## Funding

This work was supported by grants from the National Natural Science Foundation of China (82370130), the Key Medical Discipline Construction Project of the Xuhui District (SHXHZDXK202316), the Shanghai Municipal Committee of Science and Technology (24ZR1410100), the Shanghai Academic/Technology Researcher Leader Program (20XD1401000), and the Shanghai Engineering Research Center for Tumor Multitarget Gene Diagnosis (20DZ2254300). All authors obtain permission to acknowledge all those mentioned in the acknowledgements.

## Conflicts of Interest

The authors declare no conflicts of interest.

## Ethics Statement

The study was in accordance with the ethical standards formulated in the Helsinki Declaration and was approved by Institutional Review Board of Zhongshan Hospital, Fudan University. Written informed consent was obtained from each patient before being included in the study.

## Supporting information




**Supporting File 1**: advs77980‐sup‐0001‐SuppMat.docx.

**Supporting File 2**: advs77980‐sup‐0002‐SuppMat.docx.

## Data Availability

The data that support the findings of this study are available from the corresponding author upon reasonable request.
