# Supplementary material for: Single‐Cell Transcriptomic Profiling Identifies B Cell‐Intrinsic Dysregulation of Sphingolipid Biosynthesis and Could Be Improved by Inhibiting UGCG in Primary Immune Thrombocytopenia
Source: Adv Sci (Weinh). 2026 Sep 27:e77980. Online ahead of print. doi: 10.1002/advs.77980 (PMC13616311; doi:10.1002/advs.77980)
Supplement: Supplementary file 1 [file ADVS-9999-e77980-s001.docx]

**Supplementary Figure 1**

**A.** Boxplots showing the expression levels of each sample after quality control.

**B.** Boxplots showing the cluster purity (assessed by the average ROGUE value) of B-cell subsets.

**C.** Dotplots showing enriched pathways in B cells from HC and ITP samples.

**D.** UMAP plots showing the class-switch recombination (CSR) score (CSR-related signature genes: APEX1, APEX2, XRCC5, XRCC6, POLD2, and AICDA) in B-cell subsets.

**E.** Violin plots showing the CSR score in B cells. Left: grouped by disease status; right: grouped by B-cell subsets.

**Supplementary Figure 2**

**A.** UMAP plots showing the differentiation state of each B-cell subset as inferred by Monocle3.
**B–I.** Marker genes of each B-cell subset that changed as a function of pseudotime, identified using graph autocorrelation analysis with the 'graph_test' function in Monocle3.

**Supplementary Figure 3**

**A–D.** Dot plots showing upregulated ligand-receptor interactions between B-cell subsets and other cell populations. The x-axis indicates B-cell subsets and interacting cell types, and the y-axis indicates ligand-receptor pairs. Circle size represents statistical significance (P value), and color intensity represents the inferred communication probability.

**Supplementary Figure 4**

**A.** PlotKMEs visualization showing genes in each module ranked by kME.

**B.** Heat map showing correlations between gene co-expression modules. Purple indicates positive correlations between modules, whereas green indicates negative correlations.

**C.** Network view of RUNX3 target-gene enrichment pathways in B cells inferred by gene set enrichment analysis (GSEA).

**D–E.** Violin plots showing the expression levels of RUNX1 and RUNX3 in different groups.

**Supplementary Figure 5**

Bar plot showing the predicted target genes of RUNX3 ranked by regulatory score. Yellow represents positive regulation, and blue represents negative regulation.

**Supplementary Figure 6**

**A.** FACS plots showing SOX4 expression in different samples gated on CD19+ cells.
**B.** PCA plots showing the batch effect between two groups of bulk RNA-seq data before and after correction.
**C–D.** Bar plots showing the expression levels of inflammatory factors in different groups.
**E.** Full, uncropped scans of the blots shown in Figure 7.

**Supplementary Figure 7**

**A.** UMAP visualization of B cells colored according to the inferred cell-cycle phase (G1, S, and G2/M).

**B–C.** Distribution of S-phase scores (B) and G2/M-phase scores (C) across Bc_1–Bc_6.

**D.** Pie charts showing the proportions of G1-, S-, and G2/M-phase cells within each B-cell subset. Bc_5 comprised 57.0% G1-phase, 26.4% S-phase, and 16.6% G2/M-phase cells and did not show an apparent enrichment of cycling cells compared with the other major B-cell subsets.

**Supplementary Figure 8**

**A.** UMAP visualization colored by disease group (HC, ITP, and ET) .

**B.** UMAP visualization colored by individual sample identity after Harmony integration.

**C.** UMAP visualization stratified by disease group and colored according to major cell types.

**D.** Sample-level integration local inverse Simpson's index (iLISI) calculated within each major cell type using Harmony embeddings. Higher iLISI values indicate greater local mixing of cells from different samples.
