# Supplementary material for: Single‐Cell Transcriptomic Profiling Identifies B Cell‐Intrinsic Dysregulation of Sphingolipid Biosynthesis and Could Be Improved by Inhibiting UGCG in Primary Immune Thrombocytopenia
Source: Adv Sci (Weinh). 2026 Sep 27:e77980. Online ahead of print. doi: 10.1002/advs.77980 (PMC13616311; doi:10.1002/advs.77980)
Supplement: Supplementary file 2 [file ADVS-9999-e77980-s002.docx]

# Contents

| **Content of lists in this study** | |
| --- | --- |
| table1 | Primer sequences used in this study. |
| table2 | list of differetially expressed genes of B cell subsets in this study. |
| table3 | list of periphral blood cell counts in mice in this study. |
| table4 | list of genes associated with sphingolipid metabolism. |
| table5 | top TFs in Bc_5 by hdWGCNA analysis |
| table6 | list of genes regulated by RUNX3. |
| table7 | Detailed information of antibodies used in this study. |
| table8 | Cell-cycle scoring and phase distribution across B-cell subsets. |
| table9 | iLISI results across cell types in this study. |

# Table 1

| **Supplementary Table 1. Primer sequences used in this study** | | | |
| --- | --- | --- | --- |
| Species/ | Gene name | Forward Sequence (5’-3’) | Reverse Sequence (5’-3’) |
| homo sapiens | *IL32* | TGGCGGCTTATTATGAGGAGC | CTCGGCACCGTAATCCATCTC |
|  | *NKG7* | GATTGCTTTGAGCACCGATTTC | GTAGCCTGATATGATGTCCCCA |
|  | *GNLY* | CCTGTCTGACGATAGTCCAAAAA | GACCTCCCCGTCCTACACA |
|  | *CCL5* | CCAGCAGTCGTCTTTGTCAC | CTCTGGGTTGGCACACACTT |
|  | *CD69* | ATTGTCCAGGCCAATACACATT | CCTCTCTACCTGCGTATCGTTTT |
|  | *CD83* | AAGGGGCAAAATGGTTCTTTCG | GCACCTGTATGTCCCCGAG |
|  | *CXCR3* | CCACCTAGCTGTAGCAGACAC | AGGGCTCCTGCGTAGAAGTT |
|  | *ABCA7* | CCCTCAACTGGTACGAGGCTA | AGCTTCCGGGTAAAAGGTGTA |
|  | *CYFIP2* | CAACGTGGACCTGCTTGAAGA | AGTTTGTGTCAAAGTTAGCCTGG |
|  | *CD7* | GCCTCCGTCAACATCACCTG | ACCCCGTCCTCGTAGTAAATG |
|  | *MBNL3* | CCTATGACTCCATCAATTCCAGC | CGCATCAGTTTTGGGCCAAC |
|  | *ATXN2L* | GGAGCCGAAGGCATCTTGG | TCTGGAATTGTTGTAGACGCC |
|  | *TGFBR3* | TGGGGTCTCCAGACTGTTTTT | CTGCTCCATACTCTTTTCGGG |
|  | *USP34* | TGGCTACATATTCCCGCTGTC | GCTGCACTCTGTCGTAACTCC |
|  | *KLF12* | CGGCAGTCAGAGTCAAAACAG | CGGCTTCCATATCGGGATAGT |
|  | *CD22* | GCACCCTGAAACCCTCTACG | ATCAAACTTCGAGGTGTTCTTGT |
|  | *GAPDH* | GGAGCGAGATCCCTCCAAAAT | GGCTGTTGTCATACTTCTCATGG |

# Table 2

**Supplementary Table 2. List of differetially expressed genes of B cell subsets in this study.**

**top10 differentially expressed genes in B cell subsets**

| **cluster_display** | **gene** | **avg_log2FC** | **p_val** | **p_val_adj** | **pct.1** | **pct.2** | **cluster** |
| --- | --- | --- | --- | --- | --- | --- | --- |
| Bc_1 | IGHM | 1.55441713707 | 0 | 0 | 0.996 | 0.857 | 0 |
| Bc_1 | IGHD | 1.23790157641 | 1.32159310267e-299 | 2.64318620533e-296 | 0.956 | 0.769 | 0 |
| Bc_1 | CXCR4 | 1.06099642287 | 2.67887768013e-268 | 5.35775536027e-265 | 0.976 | 0.904 | 0 |
| Bc_1 | YBX3 | 1.03594524118 | 1.49670555548e-200 | 2.99341111096e-197 | 0.954 | 0.837 | 0 |
| Bc_1 | TXNIP | 0.893839756447 | 6.70812254906e-232 | 1.34162450981e-228 | 1 | 0.99 | 0 |
| Bc_1 | IL4R | 0.86390204031 | 1.32961643055e-190 | 2.6592328611e-187 | 0.873 | 0.718 | 0 |
| Bc_1 | FCER2 | 0.862340822664 | 7.76590013007e-236 | 1.55318002601e-232 | 0.904 | 0.749 | 0 |
| Bc_1 | BIRC3 | 0.785942248986 | 3.21956497027e-175 | 6.43912994053e-172 | 0.927 | 0.856 | 0 |
| Bc_1 | VPREB3 | 0.702500971792 | 3.21757603379e-161 | 6.43515206758e-158 | 0.923 | 0.822 | 0 |
| Bc_1 | SESN1 | 0.616372484773 | 8.63667780571e-145 | 1.72733556114e-141 | 0.857 | 0.738 | 0 |
| Bc_2 | CRIP1 | 1.95330662386 | 5.37606577011e-175 | 1.07521315402e-171 | 0.94 | 0.835 | 1 |
| Bc_2 | AIM2 | 0.993546894773 | 1.82648101554e-145 | 3.65296203108e-142 | 0.88 | 0.75 | 1 |
| Bc_2 | MARCKS | 0.917291048175 | 7.20121093966e-118 | 1.44024218793e-114 | 0.899 | 0.819 | 1 |
| Bc_2 | CIB1 | 0.891918017081 | 1.12450617025e-122 | 2.24901234049e-119 | 0.916 | 0.853 | 1 |
| Bc_2 | SCIMP | 0.878173754467 | 4.20347528949e-117 | 8.40695057897e-114 | 0.91 | 0.825 | 1 |
| Bc_2 | MS4A1 | 0.80532387621 | 4.69456909589e-139 | 9.38913819179e-136 | 0.996 | 0.956 | 1 |
| Bc_2 | LGALS1 | 0.80138036191 | 5.94247905351e-71 | 1.1884958107e-67 | 0.871 | 0.815 | 1 |
| Bc_2 | TNFRSF13B | 0.792311955864 | 9.47163551388e-115 | 1.89432710278e-111 | 0.835 | 0.76 | 1 |
| Bc_2 | KLF6 | 0.786083142201 | 3.66341471838e-105 | 7.32682943676e-102 | 0.983 | 0.921 | 1 |
| Bc_2 | BANK1 | 0.780790233191 | 2.35739133896e-132 | 4.71478267793e-129 | 0.981 | 0.921 | 1 |
| Bc_3 | IGLC1 | 5.51243852009 | 6.62740610337e-53 | 1.32548122067e-49 | 0.989 | 0.918 | 2 |
| Bc_3 | IGHG2 | 5.33197779459 | 7.62739654531e-82 | 1.52547930906e-78 | 0.924 | 0.805 | 2 |
| Bc_3 | IGHG1 | 4.77941609701 | 1.16188495818e-101 | 2.32376991636e-98 | 0.964 | 0.855 | 2 |
| Bc_3 | JCHAIN | 4.75086514998 | 1.24953544965e-142 | 2.4990708993e-139 | 0.993 | 0.856 | 2 |
| Bc_3 | IGHA1 | 4.58461883507 | 7.53886561096e-56 | 1.50777312219e-52 | 0.975 | 0.878 | 2 |
| Bc_3 | IGHA2 | 4.34821854141 | 3.9132039047e-17 | 7.82640780941e-14 | 0.842 | 0.794 | 2 |
| Bc_3 | IGHG4 | 4.11085015598 | 2.48377130673e-59 | 4.96754261346e-56 | 0.986 | 0.894 | 2 |
| Bc_3 | MZB1 | 3.31299558468 | 1.26128693031e-147 | 2.52257386061e-144 | 0.986 | 0.835 | 2 |
| Bc_3 | HSP90B1 | 3.01871642217 | 4.43553693456e-148 | 8.87107386911e-145 | 1 | 0.906 | 2 |
| Bc_3 | TXNDC5 | 2.95618079311 | 4.35296480144e-153 | 8.70592960287e-150 | 0.989 | 0.813 | 2 |
| Bc_4 | GNLY | 2.7889843666 | 6.05104169002e-44 | 1.210208338e-40 | 0.794 | 0.514 | 3 |
| Bc_4 | NKG7 | 2.49059235779 | 9.10396999849e-47 | 1.8207939997e-43 | 0.844 | 0.649 | 3 |
| Bc_4 | IL7R | 2.42132053612 | 2.69960149755e-72 | 5.39920299509e-69 | 0.965 | 0.736 | 3 |
| Bc_4 | CCL5 | 2.22695194467 | 1.63688895725e-42 | 3.27377791449e-39 | 0.851 | 0.698 | 3 |
| Bc_4 | IL32 | 2.0429587335 | 3.96520201038e-87 | 7.93040402076e-84 | 0.986 | 0.661 | 3 |
| Bc_4 | HCST | 1.71740966301 | 8.07943997661e-82 | 1.61588799532e-78 | 0.993 | 0.758 | 3 |
| Bc_4 | CD3G | 1.53650712393 | 4.1442009231e-84 | 8.28840184619e-81 | 0.972 | 0.638 | 3 |
| Bc_4 | GIMAP7 | 1.47805948214 | 3.48673408622e-79 | 6.97346817244e-76 | 0.972 | 0.704 | 3 |
| Bc_4 | CD3E | 1.45809568734 | 2.13887367417e-77 | 4.27774734835e-74 | 0.95 | 0.636 | 3 |
| Bc_4 | S100A4 | 1.42700795265 | 2.35266920025e-56 | 4.7053384005e-53 | 0.986 | 0.864 | 3 |
| Bc_5 | SERPINB1 | 2.02063911192 | 6.11674954291e-62 | 1.22334990858e-58 | 0.992 | 0.825 | 4 |
| Bc_5 | CDK6 | 1.91237877118 | 1.06273690941e-70 | 2.12547381881e-67 | 0.983 | 0.733 | 4 |
| Bc_5 | ANXA1 | 1.80190879171 | 7.30135828806e-63 | 1.46027165761e-59 | 0.975 | 0.713 | 4 |
| Bc_5 | SOX4 | 1.77787644687 | 6.18510990712e-67 | 1.23702198142e-63 | 0.966 | 0.681 | 4 |
| Bc_5 | CLC | 1.43104217067 | 4.99597920822e-08 | 9.99195841644e-05 | 0.664 | 0.568 | 4 |
| Bc_5 | AIF1 | 1.41901261458 | 3.44999515874e-62 | 6.89999031749e-59 | 0.975 | 0.743 | 4 |
| Bc_5 | IGFBP7 | 1.29984211647 | 1.23138829955e-71 | 2.4627765991e-68 | 0.975 | 0.605 | 4 |
| Bc_5 | NUCB2 | 1.2456239198 | 9.74744687784e-61 | 1.94948937557e-57 | 0.983 | 0.769 | 4 |
| Bc_5 | LDHB | 1.23273273086 | 1.08564295278e-50 | 2.17128590556e-47 | 0.992 | 0.855 | 4 |
| Bc_5 | EGFL7 | 1.22445537768 | 1.34623651301e-68 | 2.69247302601e-65 | 0.966 | 0.572 | 4 |
| Bc_6 | LYZ | 5.02296695188 | 3.348576712e-56 | 6.697153424e-53 | 1 | 0.846 | 5 |
| Bc_6 | S100A9 | 4.16690899726 | 6.48918898592e-56 | 1.29783779718e-52 | 1 | 0.992 | 5 |
| Bc_6 | S100A8 | 3.74091560978 | 2.02176917528e-55 | 4.04353835056e-52 | 1 | 0.98 | 5 |
| Bc_6 | S100A4 | 3.55908458518 | 2.44546354046e-55 | 4.89092708093e-52 | 1 | 0.865 | 5 |
| Bc_6 | VCAN | 3.44270246169 | 1.75075079225e-54 | 3.5015015845e-51 | 0.988 | 0.712 | 5 |
| Bc_6 | FCN1 | 3.00566173976 | 3.10433199607e-54 | 6.20866399214e-51 | 0.988 | 0.741 | 5 |
| Bc_6 | S100A6 | 2.99577002335 | 1.53005348555e-55 | 3.06010697109e-52 | 1 | 0.909 | 5 |
| Bc_6 | TYROBP | 2.96238735013 | 2.86530668064e-56 | 5.73061336128e-53 | 1 | 0.728 | 5 |
| Bc_6 | CST3 | 2.96204364952 | 3.74724414015e-54 | 7.49448828029e-51 | 0.988 | 0.737 | 5 |
| Bc_6 | MNDA | 2.91533498716 | 6.53709174513e-56 | 1.30741834903e-52 | 1 | 0.78 | 5 |

**KEGG enrichment analysis in B cell subsets**

| **Cluster** | **ID** | **Description** | **GeneRatio** | **BgRatio** | **pvalue** | **p.adjust** | **qvalue** | **geneID** | **Count** |
| --- | --- | --- | --- | --- | --- | --- | --- | --- | --- |
| Bc_1 | hsa04064 | NF-kappa B signaling pathway | 45742 | 105/8847 | 0.00346333702053 | 0.0830827021545 | 0.0710575742111 | LTB/BIRC3/TRAF5 | 3 |
| Bc_1 | hsa04668 | TNF signaling pathway | 45742 | 119/8847 | 0.00492308598911 | 0.0830827021545 | 0.0710575742111 | BIRC3/JUN/TRAF5 | 3 |
| Bc_2 | hsa04820 | Cytoskeleton in muscle cells | 45805 | 232/8847 | 0.000712646825263 | 0.0580841748207 | 0.0469743647458 | SSPN/COL4A4/VIM/ITGB1/ACTG1 | 5 |
| Bc_2 | hsa04530 | Tight junction | 45775 | 170/8847 | 0.00187932159872 | 0.0580841748207 | 0.0469743647458 | CD1C/EZR/ITGB1/ACTG1 | 4 |
| Bc_2 | hsa05412 | Arrhythmogenic right ventricular cardiomyopathy | 45744 | 86/8847 | 0.0024376306539 | 0.0580841748207 | 0.0469743647458 | SSPN/ITGB1/ACTG1 | 3 |
| Bc_2 | hsa05410 | Hypertrophic cardiomyopathy | 45744 | 99/8847 | 0.00363481308472 | 0.0580841748207 | 0.0469743647458 | SSPN/ITGB1/ACTG1 | 3 |
| Bc_3 | hsa03060 | Protein export | 22798 | 31/8847 | 5.94203259404e-08 | 3.53550939345e-06 | 3.22120714308e-06 | SEC11C/SPCS3/TMEM208/HSPA5/SPCS2/SPCS1 | 6 |
| Bc_3 | hsa05230 | Central carbon metabolism in cancer | 22737 | 71/8847 | 0.00149600208732 | 0.0545255672738 | 0.0496783142787 | IDH2/PDK1/LDHA/LDHB | 4 |
| Bc_3 | hsa04918 | Thyroid hormone synthesis | 22737 | 75/8847 | 0.00183279217727 | 0.0545255672738 | 0.0496783142787 | PDIA4/HSP90B1/HSPA5/CANX | 4 |
| Bc_3 | hsa04612 | Antigen processing and presentation | 22737 | 80/8847 | 0.00232358670134 | 0.0553013634919 | 0.050385143208 | HSPA5/CALR/CANX/PDIA3 | 4 |
| Bc_4 | hsa01230 | Biosynthesis of amino acids | 45703 | 75/8847 | 0.00693272096206 | 0.297148564763 | 0.276947675492 | GLUL/TKT | 2 |
| Bc_4 | hsa05142 | Chagas disease | 45703 | 103/8847 | 0.0127693530174 | 0.297148564763 | 0.276947675492 | GNA15/GNAQ | 2 |
| Bc_4 | hsa05146 | Amoebiasis | 45703 | 103/8847 | 0.0127693530174 | 0.297148564763 | 0.276947675492 | GNA15/GNAQ | 2 |
| Bc_4 | hsa04724 | Glutamatergic synapse | 45703 | 116/8847 | 0.016009468096 | 0.297148564763 | 0.276947675492 | GNAQ/GLUL | 2 |
| Bc_4 | hsa04071 | Sphingolipid signaling pathway | 45703 | 122/8847 | 0.0176126247088 | 0.297148564763 | 0.276947675492 | GNAQ/FCER1A | 2 |
| Bc_5 | hsa04660 | T cell receptor signaling pathway | 27242 | 122/8847 | 7.31644495127e-06 | 0.000795970605506 | 0.000596522593371 | CD3G/CD3E/CD3D/LCK/CD247/CD8A/ITK/RASGRP1 | 8 |
| Bc_5 | hsa05340 | Primary immunodeficiency | 27150 | 38/8847 | 1.4455861041e-05 | 0.000795970605506 | 0.000596522593371 | CD3E/CD3D/IL7R/LCK/CD8A | 5 |
| Bc_5 | hsa04650 | Natural killer cell mediated cytotoxicity | 27242 | 135/8847 | 1.54324362648e-05 | 0.000795970605506 | 0.000596522593371 | LCK/HCST/CD247/PRF1/KLRD1/GZMB/FCGR3A/TYROBP | 8 |
| Bc_5 | hsa04659 | Th17 cell differentiation | 27211 | 108/8847 | 3.05281221351e-05 | 0.00112343489457 | 0.000841933473622 | CD3G/CD3E/CD3D/LCK/CD247/RORA/IL6ST | 7 |

**The expression of Bc_5 makers in RNA-seq datasets**

| **gene** | **p.value** | **t.statistic** | **mean_ITP** | **mean_HC** | **log2FC** | **p.adj** | **significance** | **x** | **y.position** | **label** |
| --- | --- | --- | --- | --- | --- | --- | --- | --- | --- | --- |
| ANXA1 | 0.0113238458386 | -3.90745106879 | 367.481734067 | 18093.8333333 | -5.62168173582 | 0.0258761727376 | * | 1.5 | 35076.8 | p=0.026 * |
| CDK6 | 0.0170296875089 | -3.5138835376 | 3.20615256167 | 811 | -7.98271502784 | 0.0258761727376 | * | 1.5 | 1970.1 | p=0.026 * |
| CLC | 0.190511562058 | -1.51356995619 | 52.9208284308 | 1246.66666667 | -4.5580963132 | 0.190511562058 | ns | 1.5 | 5542.9 | p=0.191 ns |
| SERPINB1 | 0.0207009381901 | -3.33343032065 | 96.2074448667 | 40365.1666667 | -8.71274659415 | 0.0258761727376 | * | 1.5 | 84285.3 | p=0.026 * |
| SOX4 | 0.0171412019755 | -3.50700396829 | 4.34148255067 | 239.166666667 | -5.78368473549 | 0.0258761727376 | * | 1.5 | 514.8 | p=0.026 * |

# Table 3

| **Supplementary Table 3：list of periphral blood cell counts in mice in this study.** | | | |
| --- | --- | --- | --- |
| **PBC count at day0** | | | |
|  | WBC (*10^9/L) | HGB(g/L) | PLT(*10^9/L) |
| 1 | 47 | 390 | 820 |
| 2 | 16 | 140 | 700 |
| 3 | 14 | 150 | 820 |
| 4 | 9 | 150 | 980 |
| 5 | 140 | 140 | 700 |
| **PBC count at day2** | | | |
|  | WBC (*10^9/L) | HGB(g/L) | PLT(*10^9/L) |
| wt 1 | 11 | 150 | 1100 |
| wt 2 | 9 | 270 | 870 |
| wt 3 | 9 | 110 | 600 |
| treat 1 | 8 | 150 | 280 |
| treat 2 | 13 | 190 | 330 |
| treat 3 | 10 | 190 | 420 |
| placebo 1 | 17 | 200 | 250 |
| placebo 2 | 17 | 200 | 280 |
| placebo 3 | 19 | 200 | 330 |
| **PBC count at day4** | | | |
|  | WBC (*10^9/L) | HGB(g/L) | PLT(*10^9/L) |
| wt1 | 21 | 290 | 1170 |
| wt2 | 31 | 330 | 1030 |
| wt3 | 33 | 450 | 1430 |
| wt4 | 18 | 150 | 2090 |
| treat1 | 26 | 430 | 2360 |
| treat2 | 16 | 280 | 1250 |
| treat3 | 10 | 140 | 1060 |
| treat4 | 10 | 170 | 650 |
| treat5 | 11 | 150 | 280 |
| treat6 | 7 | 150 | 110 |
| treat7 | 11 | 170 | 200 |
| treat8 | 8 | 110 | 160 |
| placebo1 | 14 | 240 | 70 |
| placebo2 | 9 | 180 | 120 |
| placebo3 | 12 | 200 | 120 |
| placebo4 | 11 | 140 | 250 |
| placebo5 | 3 | 60 | 30 |
| placebo6 | 4 | 160 | 130 |
| placebo7 | 6 | 130 | 160 |
| placebo8 | 19 | 380 | 170 |
| **PBC count at day5** | | | |
|  | WBC (*10^9/L) | HGB(g/L) | PLT(*10^9/L) |
| treat1 | 6 | 130 | 250 |
| treat2 | 14 | 160 | 270 |
| treat3 | 12 | 110 | 350 |
| treat4 | 10 | 110 | 250 |
| treat5 | 13 | 130 | 240 |
| treat6 | 11 | 110 | 490 |
| treat7 | 13 | 150 | 580 |
| treat8 | 7 | 110 | 250 |
| **PBC count at day6** | | | |
|  | WBC (*10^9/L) | HGB(g/L) | PLT(*10^9/L) |
| wt1 | 10 | 130 | 1300 |
| wt2 | 12 | 140 | 1240 |
| wt3 | 13 | 140 | 890 |
| wt4 | 15 | 150 | 1060 |
| treat1 | 10 | 130 | 140 |
| treat2 | 16 | 150 | 420 |
| treat3 | 12 | 110 | 80 |
| treat4 | 15 | 130 | 720 |
| treat5 | 10 | 110 | 340 |
| treat6 | 9 | 130 | 60 |
| treat7 | lose | lose | lose |
| treat8 | 9 | 120 | 80 |
| placebo1 | 22 | 190 | 370 |
| placebo2 | 13 | 180 | 50 |
| placebo3 | 17 | 220 | 520 |
| placebo4 | 11 | 130 | 30 |
| placebo5 | 14 | 130 | 40 |
| placebo6 | 6 | 150 | 160 |
| placebo7 | 8 | 90 | 190 |
| *Note: One animal in the placebo group died on the fifth day of administration;* *All blood cell counts were measured using 10-fold diluted samples, and the measured values were subsequently multiplied by a factor of 10 to obtain the reported values.* | | | |

# Table 4

| **Supplementary Table 4: Top 10 in network string interactions.tsv ranked by MCC method** | | |
| --- | --- | --- |
| **Rank** | **Name** | **Score** |
| 1 | CERS2 | 1.54e+28 |
| 1 | SMPD1 | 1.54e+28 |
| 1 | CERS5 | 1.54e+28 |
| 1 | CERS6 | 1.54e+28 |
| 1 | ASAH1 | 1.54e+28 |
| 6 | CERS3 | 1.54e+28 |
| 6 | ACER1 | 1.54e+28 |
| 6 | CERS4 | 1.54e+28 |
| 6 | SMPD2 | 1.54e+28 |
| 6 | UGCG | 1.54e+28 |

# Table 5

| **Supplementary Table 5 top TFs in Bc_5 cells by hdWGCNA analysis** | | | | | | |
| --- | --- | --- | --- | --- | --- | --- |
| p_val | avg_log2FC | pct.1 | pct.2 | p_val_adj | cluster | gene |
| 1.06025757071e-24 | 0.741411824561 | 0.867 | 0.545 | 1.66460438602e-20 | Bc_5 | ETS1 |
| 2.25607585524e-19 | 0.721059848129 | 0.531 | 0.229 | 3.54203909272e-15 | Bc_5 | RUNX3 |
| 4.40748801348e-38 | 0.692792909496 | 0.413 | 0.087 | 6.91975618117e-34 | Bc_5 | TCF7 |
| 6.43795280847e-84 | 0.658696384704 | 0.329 | 0.02 | 1.01075859093e-79 | Bc_5 | MAF |
| 2.8172973901e-43 | 0.501179907415 | 0.294 | 0.037 | 4.42315690245e-39 | Bc_5 | RORA |
| 1.46163821095e-82 | 0.400414236696 | 0.252 | 0.01 | 2.2947719912e-78 | Bc_5 | LEF1 |
| 5.80218191709e-40 | 0.356427747027 | 0.217 | 0.021 | 9.10942560983e-36 | Bc_5 | MYBL1 |
| 1.35681295633e-13 | 0.33991188628 | 0.371 | 0.142 | 2.13019634144e-09 | Bc_5 | STAT1 |
| 2.4761833149e-12 | 0.294764822615 | 0.364 | 0.146 | 3.8876078044e-08 | Bc_5 | NFATC3 |
| 9.40404433977e-06 | 0.28454229606 | 0.804 | 0.575 | 0.147643496134 | Bc_5 | JUNB |
| 4.37632915763e-09 | 0.259344237577 | 0.399 | 0.193 | 6.87083677749e-05 | Bc_5 | KLF3 |
| 1.96156340604e-05 | -0.294272050006 | 0.189 | 0.363 | 0.307965454748 | Bc_5 | SPIB |
| 0.000219146761581 | -0.32425333793 | 0.336 | 0.47 | 1 | Bc_5 | IRF8 |
| 5.01536902029e-05 | -0.368416502431 | 0.392 | 0.544 | 0.787412936186 | Bc_5 | MEF2C |
| 8.13451703338e-05 | -0.419679602685 | 0.273 | 0.417 | 1 | Bc_5 | TCF4 |
| 1.40439409564e-05 | -0.432577168876 | 0.476 | 0.622 | 0.220489873016 | Bc_5 | POU2F2 |

# Table 6

**Supplementary Table 6: RUNX3 target genes (positive)**

postive

CX3CR1

PTP4A2

BRD2

ABCA7

CYFIP2

CD7

MBNL3

ATXN2L

USP34

PARP9

TLR10

FBRSL1

LSP1

MAP3K14

TGFBR3

TEPSIN

CBFB

GLIPR1

ITGB7

MAP4K2

MYH9

ITPR3

GPAT4

RBBP6

HECTD3

FGD2

TRIM26

AFTPH

GNB1

ABLIM1

BTN3A2

FAM53B

ZNF395

CUL4A

LIMK1

TRANK1

NADK

KLF12

MYO1C

ASNSD1

KIAA0930

SUN1

MS4A1

ARPC4

LPXN

TOMM70

GNG2

RB1

RNF219

PPP2R5A

ADGRG1

UBA1

DNM2

PLCG2

MYBL1

UFM1

STRN4

ATP11C

MAPKAPK2

NUP205

FIG4

SCRT2

SH3KBP1

RAD21

AP1B1

CLECL1

THEM4

MINK1

TRAF4

CD3E

CCSER2

TRIP4

GM2A

TADA2B

ABI1

ARF1

OAS1

ZMAT2

NCOR2

LARGE2

ADIPOR2

IL2RA

EMX2

APOL6

CYTH4

MEN1

PNP

NAXE

OXA1L

QTRT2

CAB39

PABPC4

CANT1

LGALS8

EFCAB14

PPP3CC

IQSEC1

JMJD1C

ATP11B

TULP4

IKZF3

VOPP1

OPTN

# Table 7

| **Supplementary Table 7: Detailed information of antibodies used in this study.** | |
| --- | --- |
| **antibodies** | **Catalog** |
| Donkey anti-Rabbit IgG-APC Antibody | abs20009-100ul |
| Runx3 Recombinant Rabbit mAb (S-R356) | S0B0487-25ul |
| NKG7 (E6S2A) Rabbit mAb | 84835T |
| CD83 (D8V7V) Rabbit mAb | 99075T |
| Rabbit anti-ABCA7 Polyclonal Antibody | abs146704-50ul |
| Alexa Fluor 488 Mouse anti-Human Granulysin(RB1) | 558254 |
| Rabbit anti-CD22 Recombinant Monoclonal Antibody(R113) | abs171754-25uL |
| Rabbit anti-CD69 Polyclonal Antibody | abs124226-50ul |
| Rabbit anti-ATXN2L Polyclonal Antibody(C-term) | abs108716-50ul |
| MAPKAPK-2 (D1E11) Rabbit mAb | 12155T |
| Rabbit anti-CXCR3 Polyclonal Antibody(Center) | abs100604-50ul |
| Sox-4 (B-7) FITC | sc-518016FITC |
| CD19 Beads | Miltenyi Biotec, 130-050-301 |
| PE CD19 | 555412 |

# Table 8

**Supplementary Table 8. Cell-cycle scoring and phase distribution across B-cell subsets.**

| **B-cell subset** | **Total cells** | **Mean S score** | **Median S score** | **Mean G2/M score** | **Median G2/M score** | **G1, n (%)** | **S, n (%)** | **G2/M, n (%)** | **Cycling (S+G2/M), %** |
| --- | --- | --- | --- | --- | --- | --- | --- | --- | --- |
| Bc_1 | 1501 | -0.0174 | -0.0229 | -0.0345 | -0.0403 | 813 (54.16%) | 431 (28.71%) | 257 (17.12%) | 45.84% |
| Bc_2 | 870 | -0.0236 | -0.0297 | -0.0357 | -0.0387 | 516 (59.31%) | 217 (24.94%) | 137 (15.75%) | 40.69% |
| Bc_3 | 770 | 0.0323 | -0.0194 | 0.0098 | -0.0249 | 379 (49.22%) | 244 (31.69%) | 147 (19.09%) | 50.78% |
| Bc_4 | 716 | -0.0225 | -0.0310 | -0.0312 | -0.0396 | 408 (56.98%) | 171 (23.88%) | 137 (19.13%) | 43.02% |
| Bc_5 | 193 | -0.0103 | -0.0312 | -0.0293 | -0.0418 | 110 (56.99%) | 51 (26.42%) | 32 (16.58%) | 43.01% |
| Bc_6 | 85 | -0.0473 | -0.0619 | -0.0412 | -0.0483 | 70 (82.35%) | 6 (7.06%) | 9 (10.59%) | 17.65% |

*Note: Cell-cycle scores were calculated using canonical S-phase and G2/M-phase gene signatures. Cycling cells were defined as cells assigned to the S or G2/M phase.*

# Table 9

**Supplementary Table 9. iLISI results across cell types in this study.**

| **celltype** | **n_cells** | **n_samples** | **mean_iLISI** | **median_iLISI** | **Q1_iLISI** | **Q3_iLISI** |
| --- | --- | --- | --- | --- | --- | --- |
| DC | 191 | 10 | 4.2874 | 4.2608 | 3.7695 | 4.8166 |
| Neu | 10511 | 10 | 3.4115 | 3.4332 | 2.7764 | 4.0777 |
| T cells | 14048 | 10 | 3.3056 | 3.3016 | 2.7421 | 3.8399 |
| NK cells | 3198 | 10 | 3.1378 | 3.1221 | 2.6403 | 3.6154 |
| B cells | 2471 | 10 | 2.9085 | 2.7790 | 1.9982 | 3.7672 |
| Prog | 3804 | 10 | 2.9040 | 2.7781 | 2.2137 | 3.4664 |
| Mega | 640 | 10 | 1.4774 | 1.1908 | 1.0488 | 1.6366 |

Note: iLISI values summarize sample mixing across cell types; higher values indicate stronger local sample diversity.
